# Mesenchymal stromal/stem cells promote intestinal epithelium regeneration after chemotherapy-induced damage

**DOI:** 10.1186/s13287-024-03738-9

**Published:** 2024-04-29

**Authors:** B. Yetkin-Arik, S. A. Jansen, S. Varderidou-Minasian, B. Westendorp, K.-P. Skarp, M. Altelaar, C. A. Lindemans, M. J. Lorenowicz

**Affiliations:** 1 Center for Molecular Medicine, University Medical Center Utrecht, Universiteitsweg 100, 3584 CG Utrecht, The Netherlands; 2 Regenerative Medicine Center, Uppsalalaan 8, 3584 CT Utrecht, The Netherlands; 3 Centre for Living Technologies, Alliance TU/E, WUR, UU, UMC Utrecht, Princetonlaan 6, 3584 CB Utrecht, The Netherlands; 4 Division of Pediatrics, University Medical Center Utrecht, Utrecht, The Netherlands; 5 Pediatric Stem Cell Transplantation, Princess Máxima Center for Pediatric Oncology, Utrecht, The Netherlands; 6 Division Cell Biology, Metabolism and Cancer, Department of Biomolecular Health Sciences, Faculty of Veterinary Medicine, Utrecht University, Utrecht, The Netherlands; 7 Biomolecular Mass Spectrometry and Proteomics, Bijvoet Center for Biomolecular Research, and Utrecht Institute For Pharmaceutical Sciences, Utrecht University, Utrecht, The Netherlands; 8 Biomedical Primate Research Center, Lange Kleiweg 161, 2288 GJ Rijswijk, The Netherlands

## Abstract

**Background:**

Allogeneic hematopoietic stem cell transplantation (HSCT) is a curative treatment for leukemia and a range of non-malignant disorders. The success of the therapy is hampered by occurrence of acute graft-versus-host disease (aGvHD); an inflammatory response damaging recipient organs, with gut, liver, and skin being the most susceptible. Intestinal GvHD injury is often a life-threatening complication in patients unresponsive to steroid treatment. Allogeneic mesenchymal stromal/stem cell (MSC) infusions are a promising potential treatment for steroid-resistant aGvHD. Data from our institution and others demonstrate rescue of approximately 40–50% of aGvHD patients with MSCs in Phase I, II studies and minor side effects. Although promising, better understanding of MSC mode of action and patient response to MSC-based therapy is essential to improve this lifesaving treatment.

**Methods:**

Single cell human small intestine organoids were embedded in Matrigel, grown for 5 days and treated with busulfan for 48 h. Organoids damaged by treatment with busulfan or control organoids were co-cultured with 5000, 10,000, and 50,000 MSCs for 24 h, 48 h or 7 days and the analyses such as surface area determination, proliferation and apoptosis assessment, RNA sequencing and proteomics were performed.

**Results:**

Here, we developed a 3D co-culture model of human small intestinal organoids and MSCs, which allows to study the regenerative effects of MSCs on intestinal epithelium in a more physiologically relevant setting than existing in vitro systems. Using this model we mimicked chemotherapy-mediated damage of the intestinal epithelium. The treatment with busulfan, the chemotherapeutic commonly used as conditioning regiment before the HSCT, affected pathways regulating epithelial to mesenchymal transition, proliferation, and apoptosis in small intestinal organoids, as shown by transcriptomic and proteomic analysis. The co-culture of busulfan-treated intestinal organoids with MSCs reversed the effects of busulfan on the transcriptome and proteome of intestinal epithelium, which we also confirmed by functional evaluation of proliferation and apoptosis.

**Conclusions:**

Collectively, we demonstrate that our in vitro co-culture system is a new valuable tool to facilitate the investigation of the molecular mechanisms behind the therapeutic effects of MSCs on damaged intestinal epithelium. This could benefit further optimization of the use of MSCs in HSCT patients.

**Supplementary Information:**

The online version contains supplementary material available at 10.1186/s13287-024-03738-9.

## Introduction

Allogeneic hematopoietic stem cell transplantation (HSCT) is used as a treatment for a variety of acquired and inherited disorders of the hematopoietic system, including inborn errors of metabolism, disorders of the immune system, and hematologic malignancies (e.g. leukemia and lymphoma). An essential component of HSCT is the conditioning regimen, consisting of chemotherapy and/or total body irradiation, administrated prior to hematopoietic cell infusion. The purpose of the conditioning is to target any residual leukemic disease, to provide sufficient myeloablation for engraftment and enough immunoablation to prevent rejection against the transplanted graft. However, the conditioning also contributes to the development of a major complication of allogeneic HSCT, acute graft-versus-host-disease (aGvHD), in which immune cells from the donor attack healthy recipient tissue, including liver, skin, and gut [1–3]. Especially intestinal aGvHD is a life-threatening complication. Recent findings suggest that GvHD development in the gut is enhanced by prior chemotherapy-mediated epithelial damage [4]. This was confirmed in in vitro experiments whereby chemotherapy-mediated epithelial damage increased T cell proliferation and activation [4].

First-line treatment of aGvHD is based on systemic corticosteroids that cause immunosuppression to prevent fatal disease progression [5]. However, approximately 50% of aGvHD patients become refractory to corticosteroid treatment, resulting in high morbidity and mortality rates, and low quality of life in these patients [6–8]. Many strategies have been used for a second-line treatment of steroid refractory aGvHD patients, such as Janus kinase inhibitors (JAK), anti-thymocyte globulin (ATG), and extracorporeal photopheresis (ECP) [9, 10]. Although the JAK inhibitor, ruxolitinib, has been approved by the FDA for the treatment of steroid refractory aGvHD, like other approaches this therapy results in considerable side effects, such as cytopenia and increased risk of infection. Therefore, no agent has yet been established as an optimal second-line treatment for aGvHD [10, 11].

Over the past two decades, mesenchymal stem/stromal cells (MSCs) have formed an attractive tool for cellular therapy in a wide range of clinical settings, including aGvHD [12, 13]. MSCs are multipotent progenitor cells that are present in various tissues, including bone marrow, umbilical cord, and adipose tissue and have immunomodulatory, multipotent, and regenerative characteristics [12–16]. In recent publications, MSCs have proven to be a promising treatment in steroid-refractory aGvHD patients, with a 40–50% salvage rate in Phase II clinical trials [10, 17]. The beneficial effects of MSCs were attributed mainly to their immunomodulatory capacities. However, the direct contribution of MSCs to tissue regeneration and the underlying molecular mechanisms in the treatment of steroid-refractory aGvHD remain unknown. A better understanding of the mechanisms of MSC function during rescue and repair of damaged organs and tissues may improve the efficacy of MSC therapy in GvHD patients.

Current pre-clinical models in animal studies and homogeneous cell lines are highly relevant to the field but fail to consider the high complexity and biological variation of human tissues and organs. Immortalized and human primary cell lines have enabled detailed investigation of specific cell types but do not recapitulate the cellular heterogeneity characteristics for physiological tissues. Although animal models provide a better physiological context, they are troubled by ethical concerns and translational limitations due to interspecies differences. For this purpose and to interrogate direct effects of MSC on epithelium, a suitable in vitro model is needed, which partially recapitulates the in vivo situation of intestinal epithelial damage. In the present study, we developed a co-culture model of chemotherapy-induced damage in small intestine human organoids [4, 18], self-organizing 3D mini-guts, and human bone marrow derived MSCs, and studied the regenerative aspects of MSC treatment on damaged intestinal epithelium. With the use of this co-culture model, the in vivo interplay between damaged intestinal epithelium and MSCs was mimicked, providing a micro-physiological environment relevant to post-chemotherapy injury.

## Material and methods

### Intestinal organoid culture

Human healthy duodenal organoids were cultured from biobanked frozen organoids that had been previously generated from biopsies obtained from individuals initially suspected of coeliac disease, but declared free of pathology [18, 19]. Individuals had been provided written informed consent to participate in this study according to a protocol reviewed and approved by the review board of the UMC Utrecht, the Netherlands (protocol STEM study, METC 10-402/K). To generate organoids intestinal tissues were washed and stripped of the underlying muscle layers. Tissues were chopped into approximately 5-mm pieces and further washed with cold PBS. For endoscopic biopsy samples, at least 5 biopsy samples were collected. Next, tissue fragments were incubated in 2 mmol/L EDTA cold chelation buffer (distilled water with 5.6 mmol/L Na 2 HPO4, 8.0 mmol/L KH 2 PO4, 96.2 mmol/L NaCl, 1.6 mmol/L KCl, 43.4 mmol/L sucrose, 54.9 mmol/L D -sorbitol, 0.5 mmol/L DL -dithiothreitol) for 30 min on ice. After removal of the EDTA buffer, tissue fragments were vigorously resuspended in cold chelation buffer using a 10-mL pipette to isolate intestinal crypts. The tissue fragments were allowed to settle down under normal gravity for 1 min and the supernatant was removed for inspection by inverted microscopy. The supernatants containing crypts were collected in 50-mL Falcon tubes coated with bovine serum albumin. Isolated crypts were pelleted, washed with cold chelation buffer, and centrifuged at 150–200g for 3 min to separate crypts from single cells. Crypts fragments of epithelium, or single cells were embedded in Matrigel on ice (growth factor reduced, phenol red free; BD Biosciences) and seeded in 48-well plates (500 crypts/fragments or 1000 single cells per 25uL of Matrigel per well). For maintenance of organoid cultures, single cell organoids were obtained by dissociating human small intestine organoids (passage > 7) with the use of TrypLE Express (Gibco, Grand Island, NY, USA) and washed with Advanced DMEM/F12 medium (Gibco) supplemented with 1% penicillin–streptomycin (Gibco), 1% HEPES (Gibco), and 1% glutamax (Gibco) (GF- medium). Single cell organoids were suspended in 33.33% human small intestinal organoids expansion medium (hSI-EM) consisting of GF-medium supplemented with 50% Wnt-3a conditioned-medium, 20% R-spondin-1 CM, 10% Noggin CM, EGF (50 ng/ml; Peprotech, Rocky Hill, NJ, USA), nicotinamide (10 mM; Sigma-Aldrich, Buchs, Zwitserland), N-acetyl-L-cysteine (1.25 mM; Sigma-Aldrich), B27 (Gibco), TGF-β inhibitor A83-01 (500 nM; Tocris Bioscience, Abingdon, UK), and P38 inhibitor SB202190 (10 µM; Sigma-Aldrich), and embedded in 66.66% matrigel (Corning, Bedford, MA, USA) (3.333 single cells/10 µl matrigel). Matrigel was polymerized for 15 min at 37 °C and overlaid with hSI-EM. Medium was changed every 2–3 days and Rho-kinase/ROCK inhibitor Y-27632 (10 µM; Abcam, Cambridge, UK) was added to the hSI-EM for the first 2–3 days to avoid anoikis.

### Primary cell cultures

Primary MSCs were classified as advanced therapy medicinal products (ATMPs), qualified according to standards set by the International Society of Cellular Therapy [20] and manufactured in the GMP-licensed Cell Therapy Facility (Department of Clinical Pharmacy at the UMC Utrecht), as described earlier [21–24]. Briefly, MSCs were isolated from third-party non-HLA-matched healthy bone marrow donors as approved by the Dutch Central Committee on Research Involving Human Subjects (CCMO, Biobanking bone marrow for MSC expansion, NL41015.041.12) and all samples were obtained with written informed consent from the bone marrow donor or parent/legal guardian of the donor. MSCs were grown in α-minimal essential medium (α-MEM) (Gibco) supplemented with 10% fetal bovine serum, 1% penicillin–streptomycin (Gibco), 1% L-glutamine (Gibco), basic fibroblast growth factor (bFGF) (1ng/ml; Gibco), and L-ascorbic acid phosphate (200 µM; Sigma-Aldrich) at 37 °C and 5% CO_2_. Experiments were performed with sub-confluent MSCs at passage 5–9. Description of MSC donors used in this study are listed in Additional file 1: Table S1.

### Co-cultures of small intestine organoids and MSCs

**Fig. 1:**
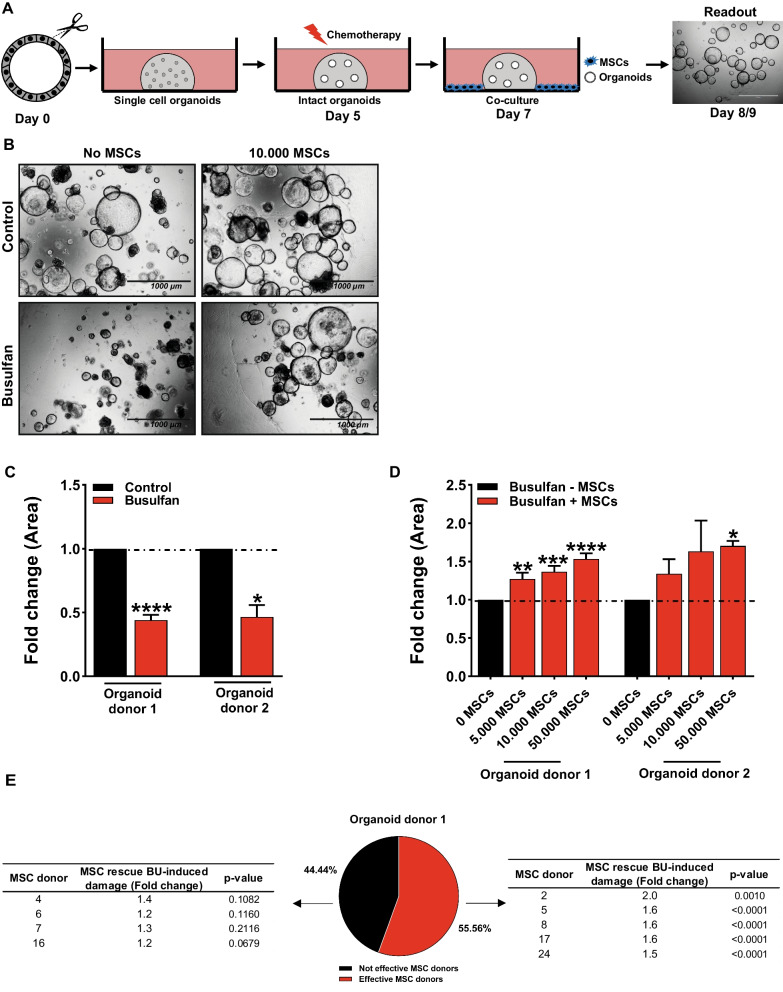
Co-culture with MSCs rescues the busulfan-induced damage of small intestine organoids. **A** Schematic overview of the in vitro co-culture model of MSCs and small intestine organoids damaged by treatment with busulfan. Single cell small intestine organoids were embedded in matrigel and grown for 5 days (day 0–5) and treated with busulfan (35 µM) for 48 h (day 5–7). Organoids damaged by treatment with busulfan or control organoids were co-cultured with 5000, 10,000, and 50,000 MSCs for 24 h or 48 h (day 7–8/9) and the surface area of organoids was measured. **B** Representative images of control organoids and organoids treated with busulfan co-cultured without or with 10,000 MSCs at 48 h after co-culture are shown. **C** Busulfan reduced the size of the organoids in organoid donor 1 and organoid donor 2. **D** Co-culture with MSCs increased the size of the organoids after treatment with 5000, 10,000, and 50,000 cells in organoid donor 1 and increased the size of the organoids after treatment with 50,000 MSCs in organoid donor 2. **E** Co-culture with 5 out of 9 tested bone marrow derived-MSC donors increased the size of organoids treated with busulfan. The quantification of surface area of the organoids was represented as fold change as compared to control. Results are shown as means ± SEM of data from 2 different organoid donors co-cultured with at least 3 MSC donors. Due to the large biological variation in organoid size, the statistical analysis of the effect of individual MSC donors on the size of busulfan-induced damaged organoids (E) was based on all evaluable individual organoids (of at least 3 organoid/matrigel droplets cultured in different wells). Scale bars, 1000 µm. * *p* < 0.05, ** *p* < 0.01, *** *p* < 0.001, and **** *p* < 0.0001 as compared to control (Kruskal–Wallis test or a Mann Whitney test)

**Fig. 2:**
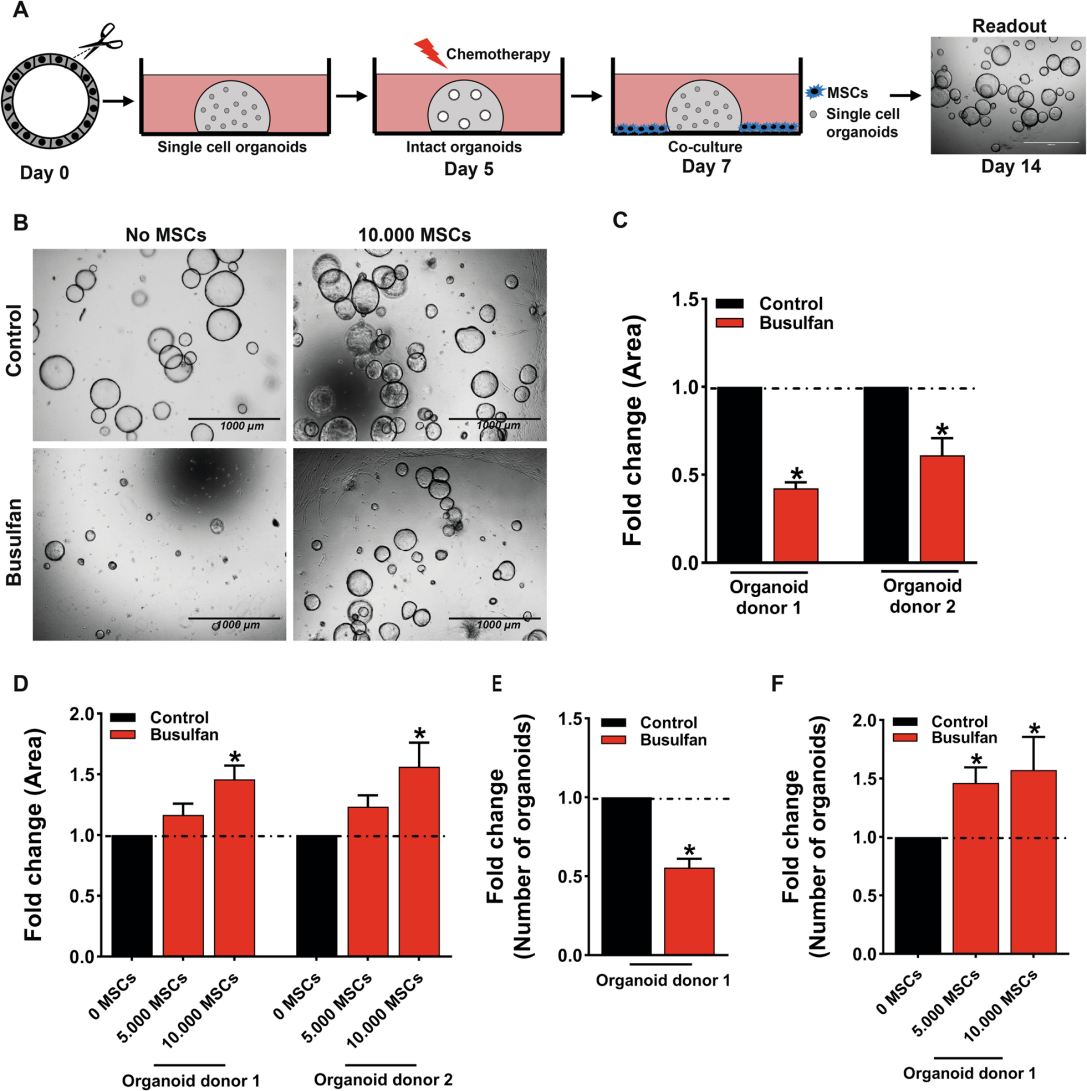
Co-culturing MSCs with single cell organoids damaged by busulfan improves intestinal organoid development. **A**. Schematic overview of the in vitro co-culture model of longer-term effects of busulfan and MSCs on small intestine organoids. Single cell small intestine organoids were embedded in matrigel and grown for 5 days (day 0–5) and treated with busulfan (35 µM) for 48 h (day 5–7). After treatment with busulfan organoids were dissociated to single cells and co-cultured with 5000 and 10,000 MSCs for 7 days (day 7–14) and the surface area and total number of organoids were measured. B. Representative images of control organoids and busulfan-treated organoids co-cultured without or with 10,000 MSCs at 7 days after co-culture are shown. **C** Busulfan reduced the size of the organoids in organoid donor 1 and organoid donor 2. **D** Co-culture with 10,000 MSCs increased the size of the organoids in organoid donor 1 and in organoid donor 2 . **E** Busulfan reduced the number of organoids in organoid donor 1. **F** Co-culturing these organoids with 5000 and 10,000 MSCs increased the number of organoids at 7 days after co-culture. The quantification of surface area of the organoids and the number of organoids was represented as fold change as compared to control. Results are shown as means ± SEM of data from 2 different organoid donors co-cultured with at least 3 MSC donors. Scale bars, 1000 µm. * *p* < 0.05 as compared to control (Kruskal–Wallis test or a Mann Whitney test)

Single cell small intestine organoids were suspended in 50% hSI-EM, embedded in 50% matrigel (Corning) and pipetted on a 48-well plate or for Transwell co-culture experiments in a 24-well plate with Transwell (Corning) inserts (500 single cells/10 µl matrigel). Matrigel was polymerized for 15 min at 37 °C and overlaid with hSI-EM supplemented with Rho-kinase/ROCK inhibitor Y-27632 (10 µM; Abcam) for the first 2 days (day 0–2). Medium was changed with a corresponding medium ratio of 1:7 MSC medium:hSI-EM every 2–3 days. Small intestine organoids were treated with busulfan (35 µM; Busilvex or TEVA) in 1:7 MSC medium: hSI-EM ratio for 48 h (day 5–7) and MSCs were co-cultured with busulfan-treated small intestine organoids at a density of 5000, 10,000, and 50,000 cells for 24 h or 48 h (day 7–8/9) (a schematic overview is shown in Fig. 1A). When using 24-well plates, these numbers were scaled according to the area (for example, 17,000 MSCs were used in 24-well plates vs. 10,000 in 48-well format). For longer incubation with MSCs, organoids were treated with 3.5 µM busulfan (Busilvex or TEVA) for 48 h (day 5–7), dissociated to single cells and co-cultured with MSCs at a density of 5000 and 10,000 cells for 7 days (day 7–14) (a schematic overview is shown in Fig. 2A). When indicated, MSCs were labeled with CellTraceTM violet dye (Thermo Fisher Scientific, Waltham, MA, USA), according to the manufacturer’s protocol. For cell sorting experiments, co-cultures of small intestine organoids and MSCs were sorted on the basis of CellTraceTM violet dye expression on a BD FACSJazz Cell Sorter (Beckton Dickinson, Franklin Lakes, NJ, USA).

### Imaging and size analysis of organoids

Phase contrast or bright field co-culture images were acquired using an EVOS FL Cell Imaging System (Thermo Fisher Scientific). The surface area (EVOS; 1.25 × and 4 × objective) and total number of organoids (EVOS; 1.25 × objective) of at least 3 organoid/matrigel droplets that were cultured in 3 different wells for every condition were quantified (representative images are shown in Figs. 1B and 2B). Organoids without an intact intestinal epithelial barrier and lumen were not quantified and were excluded from the analysis. Quantification of total number of organoids and organoid size was carried out using Fiji software [25]. The latter was analyzed and quantified automatically as well as manually.

### Cell proliferation

Cell proliferation was assessed using Click-iT™ Plus EdU flow Cytometry Assay Kit (Thermo Fisher Scientific), according to the manufacturer’s instructions. Briefly, 10 µM 5-ethynyl-2’-deoxyuridine (EDU) was added to co-cultures of small intestine organoids and MSCs and cells were incubated in a medium ratio of 1:7 MSC medium: hSI-EM for 24 h at 37 °C (day 8–9). Organoids were dissociated using TrypLE Express (Gibco) and stained with a live/dead marker Zombie Green (1:1000; Biolegend, San Diego, USA) for 15 min at room temperature. Cells were fixed with 4% paraformaldehyde for 15 min at room temperature. Cells were then permeabilized using Clickt-iT™ saponin-based permeabilization and wash reagent for 15 min and incubated in the presence of a Click-iT™ reaction cocktail containing Click-iT™ reaction buffer, CuSO_4_, fluorescent dye picolyl azide (Alexa Fluor 647), and PBS for 30 min at room temperature. Cell proliferation was determined by CytoFlex flow cytometry with 647 nm excitation (Beckman Coulter) and analyzed using CytExpert software.

### Analysis of apoptosis

Apoptotic cell death in small intestine organoids was assessed using Annexin V apoptosis detection kit (Thermo Fisher Scientific, A23204), according to the manufacturer’s instructions. Briefly, organoids were dissociated using TrypLE Express (Gibco) and stained with a live/dead marker Zombie Green (1:1000; Biolegend) for 15 min at room temperature. Subsequently, cells were washed with Annexin-binding buffer and stained using Alexa647-labeled Annexin V (1:200) for 15 min at room temperature. The apoptotic fraction of single cell organoids was detected by flow cytometry using CytoFlex flow cytometry with 647 nm excitation (Beckman Coulter) and analyzed using CytExpert software.

### RNA-sequencing and data analysis

Small intestine organoids were treated with busulfan (35 µM; Busilvex or TEVA) and co-cultured with 10,000 MSCs for 48 h at 37 °C, as described above. Co-cultures were sorted on the basis of CellTraceTM violet dye expression on a BD FACSJazz Cell Sorter (Beckton Dickinson) and RNA was isolated from single cell organoids using TRIzol LS (Thermo Fisher Scientific). RNA was processed as described previously, following the CEL-Seq2 protocol [26]. Paired-end sequencing was performed on the Illumina Nextseq500 platform, in 1 × 75 bp run mode. Reads were aligned to the hg38 human RefSeq transcriptome using Burrows–Wheeler Transform (BWA) [27]. Approximately 15 × 10^6^ reads were mapped per sample. Transcript counts were further analyzed with the R package DEseq2 [28] using default analysis parameters, and the differential gene expression between groups was assessed as shrunken log2 fold changes (LFC). Differential expression analysis was performed using DESeq2 in RStudio. Gene enrichment analysis was performed on all significantly changed genes (*P* < 0.05) with the Enrichr application using GSEA MSigDB pathways, KEGG pathways, and GO Biological Process databases [29]. Volcano plots were generated using the R package EnhancedVolcano. Heat map analysis was performed on upregulated and downregulated genes involved in the apoptosis, proliferation, and epithelial mesenchymal transition pathway (EMT) as obtained via GSEA MSigDB pathway analysis. Databases describe cellular pathways as exploited by a list of genes, which may be associated with different cellular functions in multiple pathways. Therefore, genes enriched in the top ten upregulated and downregulated pathways and also known from the literature to play an important role in the apoptosis, proliferation, and EMT pathway, were included in the heat map analysis (in Figs. 4E and 6D).

### Mass spectrometry sample preparation and protein measurement

Small intestine organoids were treated with busulfan (35 µM; Busilvex or TEVA) and co-cultured with 10,000 MSCs for 48 h at 37 °C, as described above. Co-cultures were sorted on the basis of CellTraceTM violet dye expression (Thermo Fisher Scientific) on a BD FACSJazz Cell Sorter (Beckton Dickinson) and single cell organoids were harvested in lysis buffer (1% sodium deoxycholate (SDC), 10 mM tris(2-carboxyethyl) phosphinehydrochloride (TCEP), 40 mM chloroacetamide (CAA), and 100 mM tris(hydroxymethyl)aminomethane (TRIS), (pH 8.0) ) supplemented with phosphatase inhibitor (PhosSTOP™, Roche Diagnostics) and protease inhibitor (COmplete™ mini EDTA-free; Roche Diagnostics). The lysate was sonicated using a Bioruptor® (Diagenode, Seraing, Belgium) and centrifuged at 2500 g for 10 min at 4 °C. Proteins were concentrated using bicinchoninic acid (BCA) assay kit (Waltham MA, USA), according to manufacturer’s protocol. Proteins were digested with LysC (protein-enzyme ratio 1:50) at 37 °C for 4 h and trypsin (protein-enzyme ratio 1:50) at 37 °C for 16 h. After digestion, peptides were desalted using 1cc Sep-Pak C18 cartridges (Waters Corporation), dried in vacuum and resuspended in 50 mM triethylammonium bicarbonate at a final concentration of 5 mg/mL.

Orbitrap Fusion™ Lumos™ Mass Spectrometer (Thermo Fisher Scientific) coupled to an Agilent 1290 HPLC system (Agilent Technologies, Santa Clara, California, USA) was used for protein measurements. A double-frit trap column of 20 mm × 100 μm inner diameter (ReproSil C18, Dr Maisch GmbH, Ammerbuch, Germany) and analytical column of 40 cm × 50 μm inner diameter (ReproSil Pur C18-AQ, Dr Maisch GmbH, Ammerbuch, Germany) was used. Trapping was performed at 5 μl/min in 0.1 M acetic acid in H_2_O for 10 min and analytical separation was performed at 300 nl/min for 2 h by increasing the concentration of 0.1 M acetic acid in 80% acetonitrile (*v*/*v*). Mass spectrometer was operated in data-dependent mode, automatically switching between MS and MS/MS and full-scan was acquired at m/z 350–1500 with a resolution of 60,000 FHMW, automatic gain control (AGC) target of 200,000, and a maximum injection time of 50 ms. For each scan the most intense precursors above 5000 threshold were fragmented by higher-energy collisional dissociation using a collision energy of 38%, isolation window of 1.2 Da, a resolution of 30,000, maximum injection time of 115 ms, and an activation time of 0.1 ms. Fragment ion analysis was performed on Orbitrap with resolution of 60,000 FHMW and a low mass cut-off setting of 120 m/z.

### Mass spectrometry data processing and visualization

Proteome Discoverer software (version 2.2, Thermo Fisher Scientific) was used for protein quantification. Spectra were searched using the Swissprot database (version 2014_08) with the search engine Sequest HT. Searches were carried out using the following settings: trypsin as enzyme, maximum of two missed cleavages, taxonomy for homo sapiens, and 10 ppm precursor mass tolerance with 0.6 Da fragment mass tolerance. Carbamidomethylation on cysteine residues (+ 57.021 Da) was set to fixed modification. Dynamic modifications were set for oxidation of methionine residues (+ 15.995 Da), N termini acetylation (+ 42.011 Da) and Met-loss (− 131.040 Da) and Met-loss (− 89.030 Da). Percolator was set to FDR below 1%.

The open software PERSEUS was used for statistical and bioinformatics analysis and to generate figures. Cut-off values were set to *p* value ≤ 0.1 and ≥ 1.5-fold change differences. Enrichr (Mayaanlab.com) was used for functional analysis to identify GO terms. Similar to the heat map analysis of the RNA sequencing data, proteins enriched in the top ten upregulated and downregulated pathways, which were previously reported to play an important role in the apoptosis, proliferation, and EMT pathway, were also included in the heat map analysis (in Fig. 5D and Additional file 1: Fig. S5C).

### Statistical analysis

All experiments were performed with human small intestine organoids derived from two healthy donors and co-cultured with at least 3 MSC donors and were performed in triplicate. All data were expressed as mean ± standard error of the mean (SEM). GraphPad Prism 9 software was used to assess statistical significance by a Kruskal–Wallis test or a Mann Whitney test. Statistical significance was defined as * *p* < 0.05, ** *p* < 0.01, *** *p* < 0.001. To correct for differences between donors, factor correction, as described previously [30], was used for flow cytometry data.

## Results

### Co-culture with MSCs rescues the busulfan-induced damage of small intestine organoids

To understand the mechanisms of MSC function during rescue and repair of damaged organs and tissues in transplanted patients, we developed an in vitro co-culture model of chemotherapy damaged small intestinal organoids and MSCs (a schematic overview is shown in Fig. 1A). Single cell small intestinal organoids were embedded in matrigel, grown for 5 days (day 0–5), and treated with busulfan for 48 h (day 5–7). Organoids damaged by busulfan or control organoids were co-cultured with 5000, 10,000, and 50,000 MSCs for 24 h or 48 h (day 7–8/9) and the surface area and total number of organoids were assessed (representative images are shown in Fig. 1B). Treatment with busulfan reduced the size of the organoids by 2.3-fold and 2.2-fold in organoid donor 1 and organoid donor 2 (Fig. 1C), respectively, as compared to control, but did not affect the number of organoids (Additional file 1: Fig. S1B). Co-culture with MSCs increased the size of the busulfan-treated organoids in organoid donor 1 in a MSC density-dependent manner, by 1.3-fold, 1.4-fold, and 1.6-fold after treatment with 5000, 10,000, and 50,000 cells, respectively and increased the size of the organoids by 1.7-fold after treatment with 50,000 cells in organoid donor 2 (Fig. 1D). Co-culture of control organoids with MSCs increased the size of donor 1 organoids by 1.3-fold after treatment with 50,000 cells but did not show any effect with organoid donor 2 (Additional file 1: Fig. S1A).

These findings indicate that busulfan decreases the size of small intestinal organoids but does not affect their number. In addition, our results suggest that MSCs can rescue the busulfan-induced damage of small intestinal organoids.

### Efficacy of MSC rescue of busulfan-induced damage of small intestine organoids is MSC donor-dependent

Although MSCs have proven to be a promising treatment in steroid-refractory aGvHD patients, the success of this therapy is often limited due to donor variations [17]. For a better clinical application of MSCs in aGvHD patients, in vitro donor prediction models may overcome donor-dependent limitations and improve the efficacy of MSC therapy in these patients. For this purpose, the effect of 9 different bone marrow-derived MSC donors were analyzed in our in vitro co-culture model of MSCs and busulfan-treated small intestine organoids (Additional file 1: Table S1). Co-culture with 5 out of 9 MSC donors significantly increased the size of donor 1 organoids (Fig. 1E, Additional file 1: Fig. S1C and D) and 4 out of the 9 MSC donors showed no effect (Fig. 1E, Additional file 1: Fig. S1E and F). To test whether the rescue by the effective MSC donors was organoid donor-dependent, we analyzed 4 out of the 5 effective MSC donors in organoid donor 2 after damage by busulfan. Similarly, as in organoid donor 1, these MSC donors increased the size of the busulfan-treated donor 2 organoids (Additional file 1: Table S1).

Overall, our findings suggest that MSCs are able to rescue busulfan-induced damage in small intestine organoids, but their efficacy is MSC donor-dependent.

### Co-culturing MSCs with single cell organoids damaged by busulfan improves intestine organoid development

Busulfan is a DNA alkylating chemotherapeutic agent that affects cell growth and viability by interfering with DNA replication and transcription of RNA [31]. To test whether MSCs can rescue the longer-term effects of busulfan-induced damage in small intestine organoids, organoids were co-cultured with MSCs for 7 days after the busulfan treatment (a schematic overview is shown in Fig. 2A). Similarly, as for the investigation of short-term effects of busulfan, first single cell small intestine organoids were embedded in matrigel and grown for 5 days (day 0–5), followed by 48 h (day 5–7) treatment with busulfan. Subsequently, organoids were dissociated to single cells (day 7) and co-cultured with 5000 and 10,000 MSCs for 7 days (day 7–14) and the surface area and total number of organoids were assessed at day 14 (representative images are shown in Fig. 2B). Busulfan treatment reduced the size of the organoids by 2.4-fold and 1.6-fold in organoid donor 1 and organoid donor 2 (Fig. 2C), respectively, and the number of organoids by 1.8-fold in organoid donor 1 (Fig. 2E) as compared to control. The size of busulfan-treated organoids increased by approximately 1.5 fold in both tested organoid donors (Fig. 2D), after 7 days of co-culture with 10,000 MSCs. Long co-culture with MSCs resulted also in an increase of size in control organoids (Additional file 1: Fig. S2A and B). In addition, MSCs increased the number of organoids reconstituted after busulfan treatment by approximately 1.5 fold in organoid donor 1 after 7 days of co-culture with MSCs (Fig. 2F), but did not affect the number of control organoids (Additional file 1: Fig. S2C).

Taken together, these findings indicate that busulfan-induced long-term decrease of the size and the number of small intestinal organoids grown from single cells. In addition, our data demonstrate that MSCs increase the size of both control and busulfan-treated organoids, but stimulate the development of organoids specifically after busulfan-mediated damage.

### The MSC secretome contributes to the MSC-mediated rescue of busulfan-induced damage of small intestine organoids

**Fig. 3:**
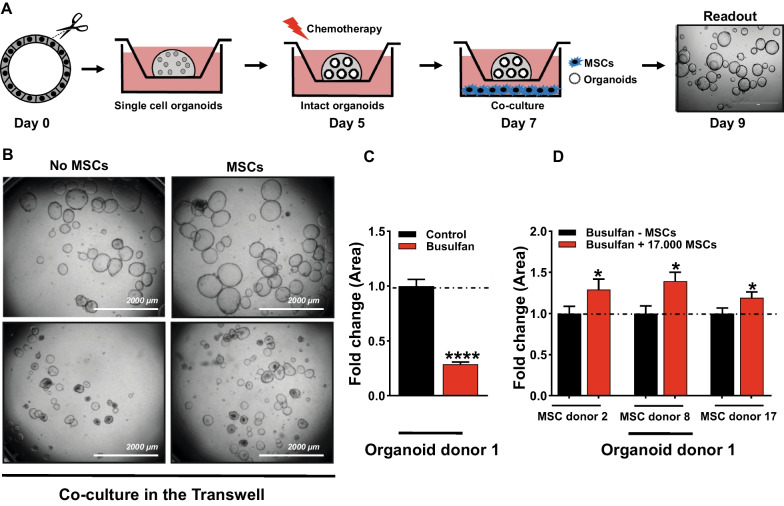
MSC secretome contributes to the rescue of busulfan-induced damage of small intestine organoids. **A** Schematic overview of the in vitro co-culture model of MSCs and small intestine organoids damaged by treatment with busulfan in a Transwell insert system. Single cell small intestine organoids were embedded in matrigel, grown for 5 days (day 0–5) in the Transwell insert, and treated with busulfan for 48 h (day 5–7). Organoids damaged by busulfan were co-cultured with 17,000 MSCs grown in the lower compartment of the Transwell system for 48 h (day 7–9) and the surface area of organoids were assessed at day 9. **B** Representative images of control organoids and organoids treated with busulfan co-cultured in Transwell system without or with 17,000 MSCs at 48 h after co-culture are shown. **C** Busulfan reduced the size of the donor 1 organoids. **D** Co-culture with MSCs in the Transwell system increased the size of the donor 1 organoids. The quantification of surface area of the organoids was represented as fold change as compared to control. Results are shown as means ± SEM of data from organoid donor 1. Due to the large biological variation in organoid size, the statistical analysis of the effect of individual MSC donors on the size of busulfan-induced damaged organoids was based on all evaluable individual organoids (1 organoid/matrigel droplet cultured in different wells in duplicate). Scale bars, 2000 µm. * *p* < 0.05, and **** *p* < 0.0001 as compared to control (Kruskal–Wallis test or a Mann Whitney test or one-way ANOVA)

Growing evidence demonstrates that beneficial effects of MSCs are derived, at least in part, from their secretome [15, 16]. To understand whether the MSC-mediated rescue of busulfan-induced damage of small intestine organoids is controlled by paracrine signalling the organoids were co-cultured with MSCs in a Transwell insert system (a schematic overview is shown in Fig. 3A.). Single cell small intestine organoids were embedded in matrigel, grown for 5 days (day 0–5) in the Transwell insert, and treated with busulfan for 48 h (day 5–7). Organoids damaged by busulfan were co-cultured with 17,000 MSCs of three different MSC donors grown in the lower compartment of the Transwell system for 48 h (day 7–9) and the surface area of organoids were assessed at day 9 (representative images are shown in Fig. 3B). Treatment with busulfan reduced the size of donor 1 organoids by 2.8-fold (Fig. 3C), as compared to control. Co-culture with MSC donor 2, 8 and 17 increased the size of the busulfan-treated organoids by 1.3-fold, 1.4-fold and 1.2-fold respectively (Fig. 3D).

Taken together, these findings indicate that MSC secretome contributes to the MSC-mediated increase in size of busulfan-treated organoids.

### Epithelial mesenchymal transition, apoptosis, and proliferation are key signaling pathways driving busulfan-induced intestinal epithelium damage

**Fig. 4:**
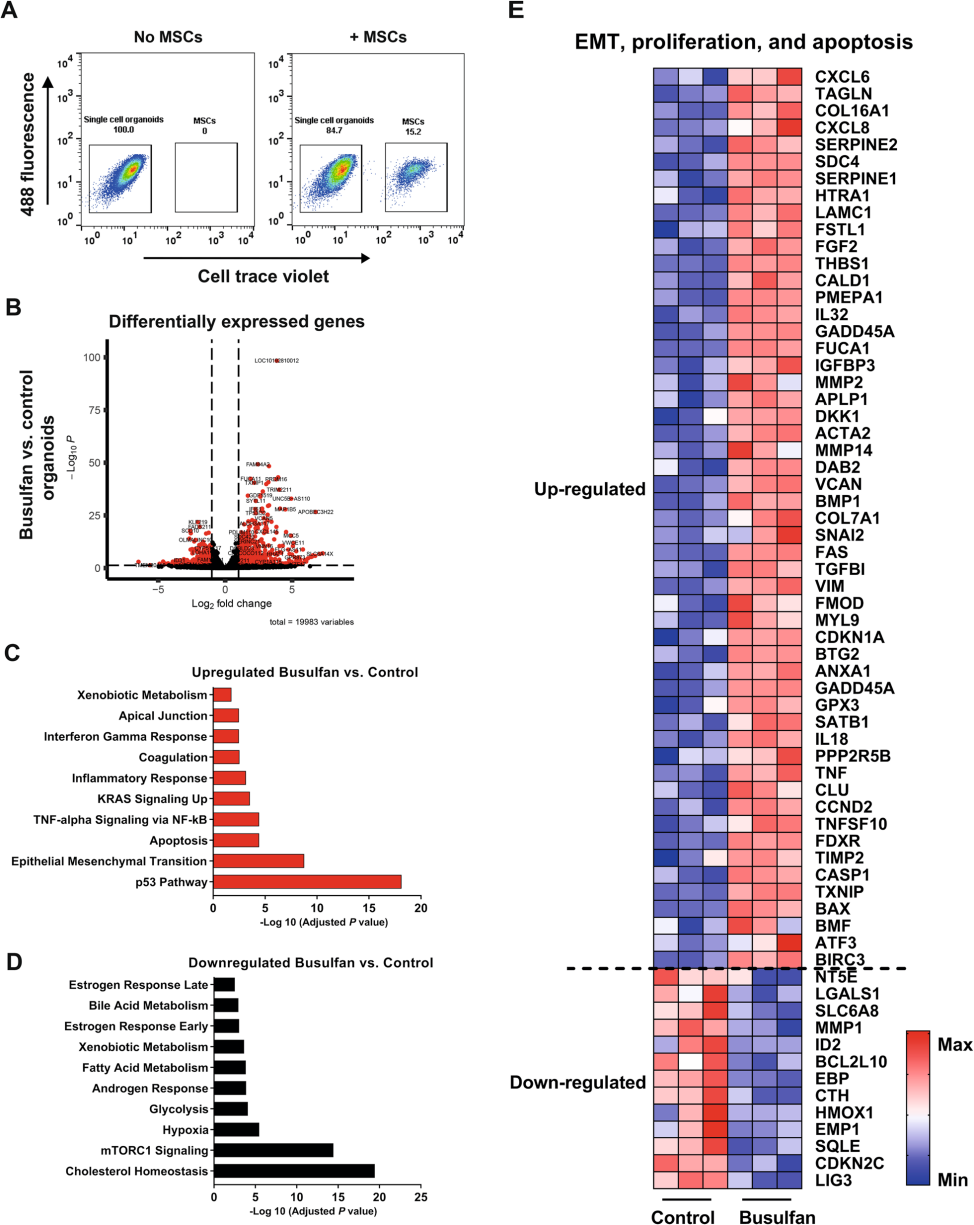
Transcriptomic analysis of busulfan-treated small intestine organoids. **A** Busulfan-treated organoids or control organoids were co-cultured with CellTraceTM violet dye-labelled MSCs for 48 h and single cell small intestine organoid were isolated from these co-cultures by FACS sorting. **B** DE analysis between control and busulfan-treated small intestine organoids (red dots indicate significant genes; *p* value < 0.05) and the accompanying upregulated (**C)** and downregulated (**D**) pathways. **E** Heat map of the differentially expressed genes involved in the EMT, proliferation, and apoptosis pathway in control organoids and busulfan-treated organoids is shown (N = 3)

**Fig. 5:**
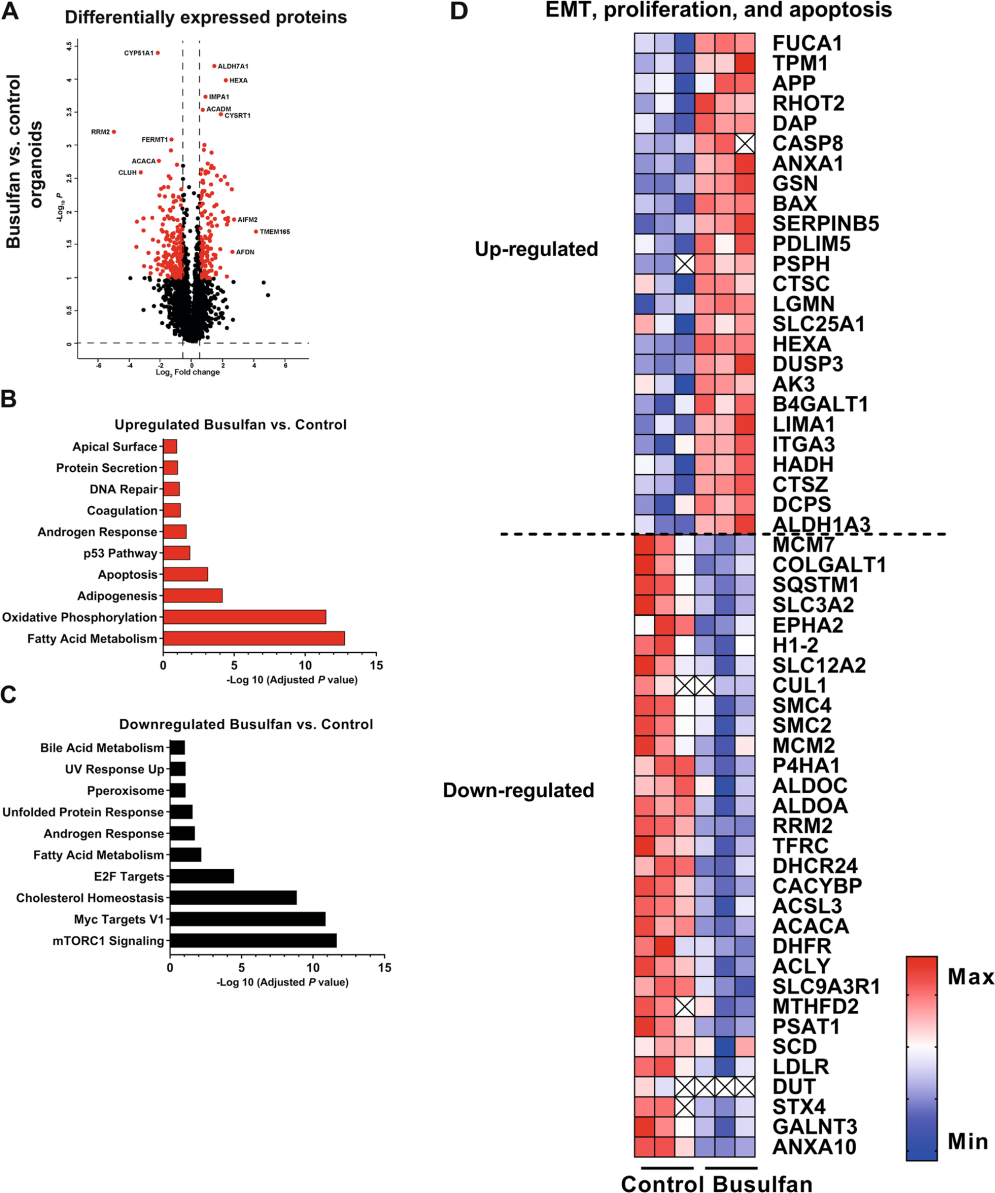
Proteomic analysis of busulfan-treated small intestine organoids. **A** DE analysis between control and busulfan-treated small intestine organoids (red dots indicate significant proteins; adjusted *p* value ≤ 0.1 and ≥ 1.5-fold change differences) and the accompanying upregulated (**B**) and downregulated (**C**) pathways are shown. **D** Heat map of the differentially expressed proteins involved in the EMT, proliferation, and apoptosis pathway in control organoids and busulfan-treated organoids is shown (N = 3)

To identify the molecular pathways controlling the MSC-mediated rescue of small intestine organoids damaged by busulfan, we performed RNA sequencing and proteomic analysis of organoids from the co-culture model. MSCs were labeled with CellTraceTM violet dye and co-cultured with control or busulfan-treated organoids for 48 h, as described above (Fig. 1A). Co-cultures were sorted on the basis of CellTraceTM violet dye expression (Fig. 4A) and RNA and proteins were isolated from single cell organoids and gene and protein expression patterns were analyzed using RNA sequencing and proteomics, respectively. Differential expression (DE) analysis showed 1734 upregulated and 1550 downregulated genes in busulfan-treated organoids as compared to control (Fig. 4B). Among the genes upregulated in busulfan-treated organoids the most strongly enriched were those regulating epithelial to mesenchymal transition, apoptosis, and p53 pathway (Fig. 4C). Among the genes downregulated in busulfan-treated organoids were regulators of mammalian target of rapamycin 1 (mTORC1) signaling and cholesterol homeostasis (Fig. 4D). Next, we characterized differences in protein expression profiles between control organoids and busulfan-treated small intestine organoids by DE analysis using the same criteria as outlined in the RNA sequencing analysis (Fig. 5A). DE analysis revealed 130 upregulated and 188 downregulated proteins in busulfan-treated organoids as compared to control. Busulfan treatment resulted in the upregulation of proteins controlling apoptosis, adipogenesis, oxidative phosphorylation, and fatty acid metabolism pathway (Fig. 5B) and downregulation of proteins controlling cholesterol homeostasis, mTORC1 signaling and myc targets V1 pathway (Fig. 5C). As both transcriptomic and proteomic analysis demonstrated that next to affecting important pathways, such as EMT and apoptosis, busulfan also had an effect on pathways regulating proliferation of intestinal epithelium, such as mTORC1 [32], we summarized significantly up- and downregulated genes and proteins involved in these pathways in a heat map analysis (Figs. 4E and 5D, respectively).

Taken together, these data indicate that the apoptosis, proliferation, and EMT pathways play an important role in the mode of action of busulfan on small intestine epithelium.

### MSCs reverse the effects of busulfan on the transcriptome and proteome in the intestinal epithelium

**Fig. 6:**
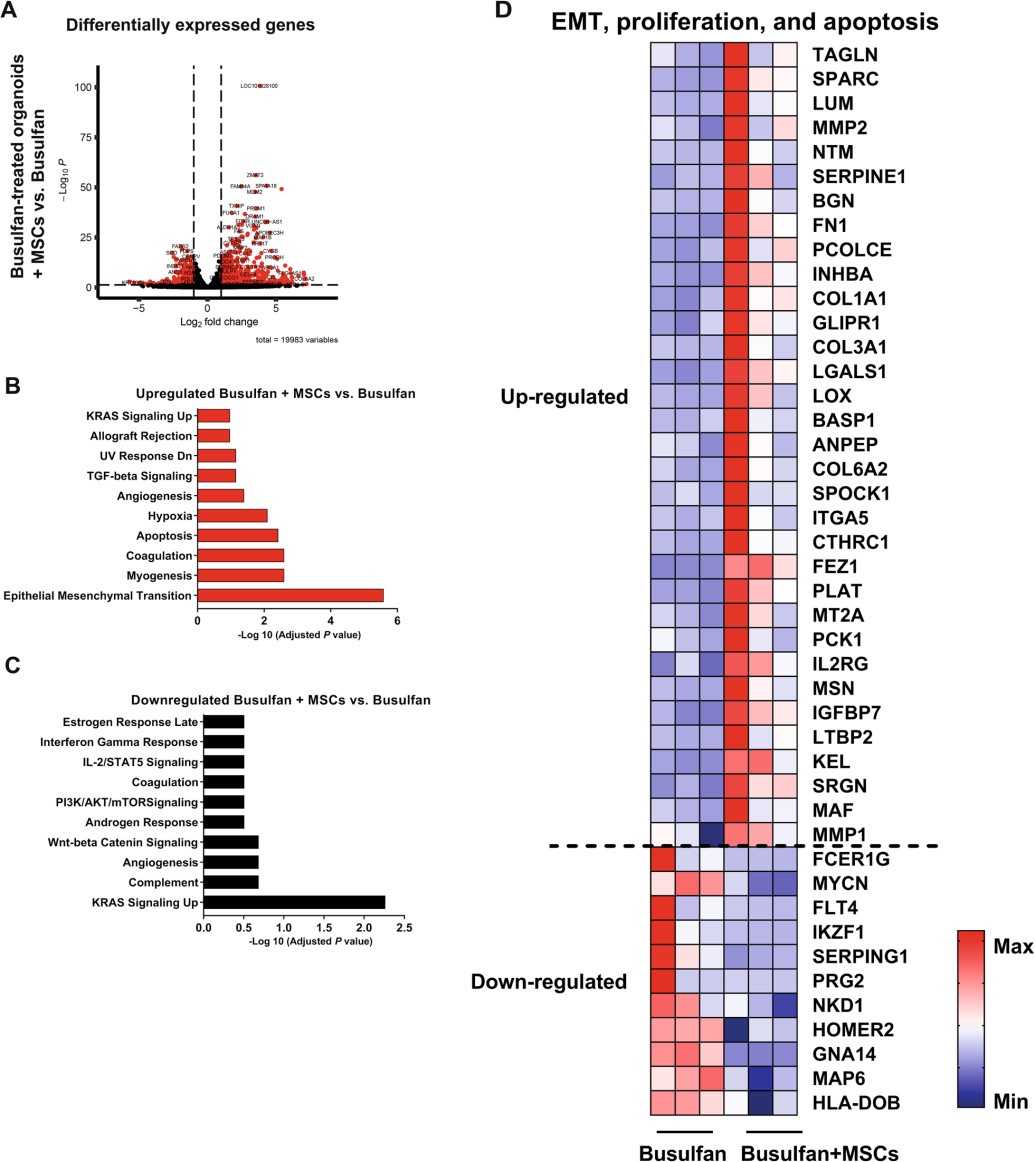
Transcriptomic analysis of busulfan-treated small intestine organoids co-cultured with and without MSCs. **A** DE analysis between busulfan-treated organoids co-cultured with 10,000 MSCs and without MSCs (red dots indicate significant genes; *p* value < 0.05) and the accompanying upregulated (**B**) and downregulated (**C**) pathways are shown. **D** Heat map of the differentially expressed genes involved in the EMT, proliferation, and apoptosis pathway in busulfan-treated organoids co-cultured with and without MSCs is shown (N = 3)

**Fig. 7:**
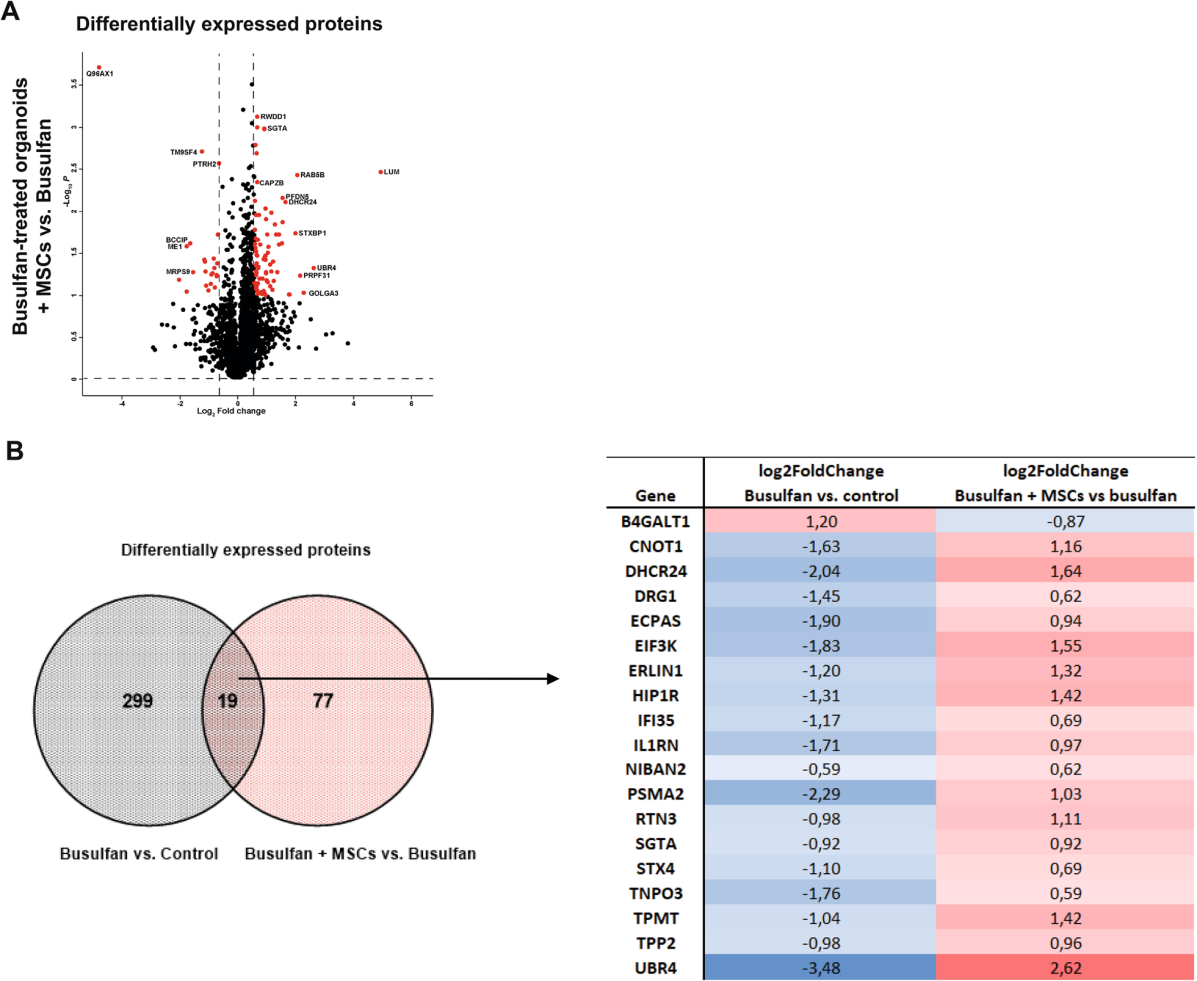
Proteomic analysis of busulfan-treated small intestine organoids co-cultured with and without MSCs. **A** DE analysis between busulfan-treated organoids co-cultured with 10,000 MSCs and without MSCs (red dots indicate significant proteins; adjusted *p* value ≤ 0.1 and ≥ 1.5-fold change differences). **B** Venn diagram of the DE proteins in busulfan-treated organoids as compared to control (grey circle) and of DE proteins in busulfan-treated organoids co-cultured with and without MSCs (red circle) and the accompanying heat map analysis is shown

To test whether MSCs can reverse the effects of busulfan on the intestinal epithelium, we characterized differences in gene and protein expression profiles between busulfan-treated organoids that were co-cultured with or without MSCs. DE analysis showed 123 upregulated and 93 downregulated genes (Fig. 6A) and 76 upregulated and 22 downregulated proteins (Fig. 7A) in organoids damaged with busulfan and co-cultured with MSCs as compared to damaged organoids without co-culture. Among genes upregulated in co-cultures of busulfan-treated organoids and MSCs, GSEA MSigDB pathway analysis showed enrichment for apoptosis and EMT pathways (Fig. 6B). The most strongly enriched pathway among downregulated genes in these co-cultures was Kirsten rat sarcoma viral (KRAS) signaling (Fig. 6C). The proteome analysis revealed the p53 and mTORC1 signaling as pathways predominantly controlled by DE proteins after co-culture of busulfan-treated organoids with MSCs (Additional file 1: Fig. S5A and B). Control intestinal organoids co-cultured with MSCs showed 124 upregulated and 186 downregulated genes (Additional file 1: Fig. S3A) and 14 upregulated and 42 downregulated proteins (Additional file 1: Fig. S4A) as compared to control organoids without MSC co-culture. The signaling pathways regulated by DE genes and proteins in control intestinal organoids co-cultured with MSCs are listed in Additional file 1: Figs. S3B and C, S4B and C, respectively. As both transcriptomic and proteomic analysis indicate that co-culture with MSCs has an effect on the EMT, apoptosis, and proliferation pathways, which are also affected by busulfan in intestinal epithelium, we summarized the genes and proteins involved in the regulation of these pathways in a heat map analysis (Fig. 6D and Additional file 1: Fig. S5C, respectively).

To gain comprehensive insights into the mode of action of MSCs in intestinal organoids treated with busulfan, we examined whether any of the significant proteins that were upregulated or downregulated by busulfan treatment were involved in MSC-mediated rescue. From the 318 significant proteins that were differentially regulated after busulfan treatment, the expression of 19 proteins was reversed by co-culture with MSCs (Fig. 7B). Interestingly, these proteins affected by MSCs were previously described to play an important role in regulating the apoptosis and/or proliferation pathway [33–50], the two most significantly affected pathways by busulfan (Fig. 5C and D).

Taken together, these findings indicate that busulfan affects pathways regulating EMT, apoptosis, and proliferation in intestinal organoids and that MSCs reverse these effects of busulfan on transcriptome and proteome of intestinal epithelium.

### MSCs promote regeneration of busulfan-induced damage in intestinal epithelium by regulating the proliferation and apoptosis pathways

**Fig. 8:**
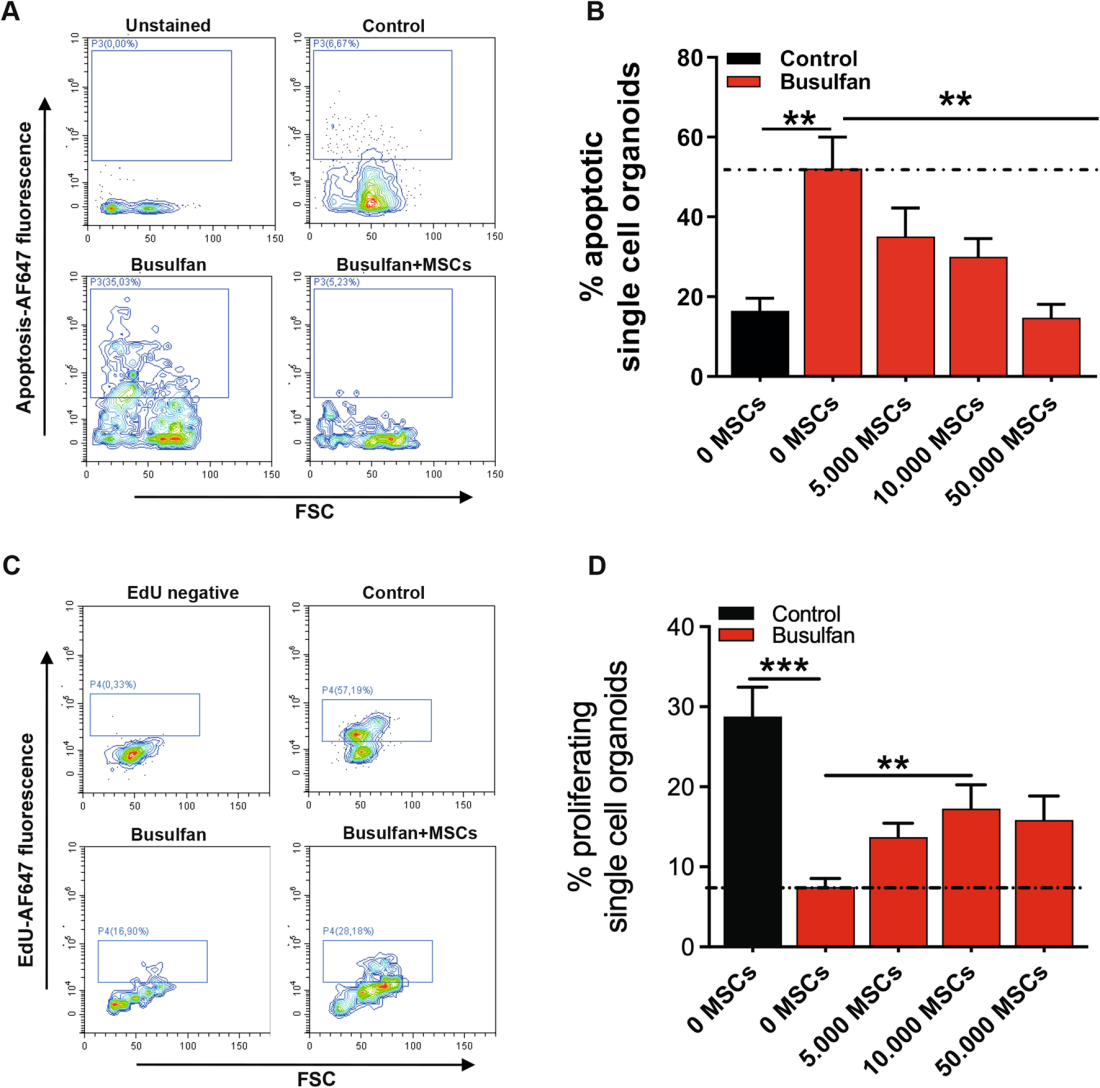
MSCs promote regeneration of busulfan-induced damage in intestinal epithelium by regulating the proliferation and apoptosis pathways. **A** Apoptotic cell death analysis by flow cytometry, of busulfan-treated single cell small intestine organoids stained with Alexa Fluor (AF) 647-conjugated annexin V after co-culture with or without MSCs. **B **Effects of co-culturing busulfan-treated organoids with 0, 5000, 10,000, and 50,000 MSCs on the percentage of apoptotic single cell organoids are shown. **C** Proliferation analysis by flow cytometry, of busulfan-treated single cell small intestine organoids stained with EdU antibody after co-culture with or without MSCs. **D** Effects of co-culturing busulfan-treated organoids with 0, 5000, 10,000, and 50,000 MSCs on the percentage of proliferating single cell organoids are shown. Results are shown as means ± SEM of organoid donor 1 and organoid donor 2 co-cultured with at least 3 MSC donors. ** *p* < 0.01 and *** *p* < 0.001 as compared to control (Kruskal–Wallis test)

Our transcriptomic and proteomic analysis indicated proliferation and apoptosis as pathways important for MSC-mediated regeneration of small intestine organoids damaged by busulfan. To verify this on a functional level, apoptosis and proliferation in busulfan-treated organoids was evaluated after co-culture with MSCs using FACS analysis. Busulfan increased the percentage of apoptotic single cell intestine organoids from 16.5 to 52.1% (Fig. 8A, B and Additional file 1: Fig. S6A). Co-culture of these organoids with MSCs decreased the percentage of apoptotic single cell intestine organoids in a cell density-dependent manner from 52.1 to 35.1%, 30%, and 14.8% after treatment with 5000, 10,000, and 50,000 cells, respectively (Fig. 8B). Co-culture of MSCs with control organoids decreased the percentage of apoptotic single cell small intestinal organoids after treatment with 50,000 cells, but did not show any effect after treatment with 5000 and 10,000 cells (Additional file 1: Fig. S6B). Next, we tested whether MSCs rescue busulfan-induced intestinal damage by stimulating cell proliferation. Busulfan decreased the percentage of proliferating single cell intestinal organoids from 28.8 to 7.5% and co-culture with MSCs increased these percentages to 14.9% after addition of 10,000 cells (Fig. 8C, D and Additional file 1: Fig. S7A). Co-culture of control organoids with MSCs did not have any effect on the percentage of proliferating single cell organoids (Additional file 1:  Fig. S8B). To test whether co-cultures of MSCs and control organoids or busulfan-treated organoids affect the viability of MSCs, the percentage of live cells in MSCs isolated from the co-cultures was determined. As shown in the Additional file 1: Fig. S7C, MSCs remained fully viable in our co-culture system.

Taken together, these data show that MSC promote the regeneration of small intestine organoids after busulfan-mediated damage by inhibition of intestinal epithelium apoptosis and induction of proliferation.

## Discussion

Allogeneic MSC infusion is a promising treatment for acute steroid refractory GvHD. However, it is still poorly understood what the exact mechanism of action of MSCs is and why only 50% of aGvHD patients respond to MSC therapy. While previous studies mainly focused on immunomodulatory capacities of MSCs, very little is known regarding the regenerative properties of MSCs towards organs, such as the intestine, which is the most affected in GvHD and in chemotherapy-mediated damage. Targeting and improving the regeneration is increasingly considered as an essential component in the design of the future and/or advancing of the current treatments for GvHD. Here, we developed an advanced in vitro co-culture model of chemotherapy-treated small intestine organoids and MSCs, which allowed us to study the regenerative effects of MSCs on intestinal epithelium. Transcriptomic and proteomic analysis performed using this model revealed that chemotherapy (busulfan) treatment affected pathways regulating EMT, proliferation, and apoptosis in small intestine organoids. The co-culture of these organoids with MSCs partially reversed the effects of busulfan. These results were further corroborated by functional evaluation of small intestine organoid proliferation and apoptosis.

Our data demonstrate that MSCs from 5 out of 9 donors tested in our co-culture system strongly increased intestinal organoid size after busulfan-mediated damage, while the 4 remaining MSC donors showed no effect. This rescue of intestinal epithelium by 50 percent of tested MSCs donors corresponds closely with the percentages of GvHD patients rescued by MSC therapy in clinical treatment of GvHD and may suggest that our co-culture system models intraindividual in vivo responses to MSC therapy [17]. However, before our results can be extrapolated to the patient response in GvHD, several points need to be still addressed. Firstly, in our co-culture model we have not included immune cells, which are main actors in the destruction of epithelium during GvHD and probably also have their individual susceptibility to MSCs. However, the interaction between MSCs and immune cells has been extensively studied before and the addition of a new cell type to our co-culture system will bring another level of complexity. Nevertheless, it is the next important step in creating an in vitro platform, that mimics the in vivo environment of GvHD. Secondly, we analyzed the effects of MSCs on intestinal epithelium, which is only one of the tissues affected in GvHD. However, the clinical outcome is determined by all the tissues damaged by transplant procedures and affected in GvHD patients. Similar co-culture systems using epithelial cells from the liver, skin, or other organs may need to be tested for a better overview of the regenerative aspect of MSC therapy. Thirdly, as the in vivo environment may influence the regenerative potency of MSCs, pre-conditioning of MSCs with pro-inflammatory cytokines released in GvHD and testing their effects on intestinal epithelium in our model should be considered [5, 51].

Busulfan is an alkylating chemotherapeutic agent that prevents cell division [52, 53]. However, its potential mechanism of action is very intricate and probably involves multiple biological processes, such as disruption of cellular redox equilibrium, proliferation, apoptosis, and autophagy [54, 55]. We investigated short- and long-term busulfan-mediated damage and the corresponding MSC-mediated rescue of human small intestine epithelium in our co-culture system. Short-term culture of intestinal organoids after treatment with busulfan resulted in the reduction of their size and MSCs reversed this effect. However, there was no detectable change in the organoid number after treatment with busulfan followed by short-term co-culture with MSCs. Our proteomic and transcriptomic analysis demonstrated the regulation of genes controlling apoptosis and proliferation after 48 h incubation with busulfan. This suggests relatively rapid induction of cell death and inhibition of cell proliferation by busulfan, resulting in a smaller size of organoids, but not immediate damage of the whole intestinal epithelium. The pro-apoptotic and anti-proliferative effects of busulfan were relatively rapidly reversed by the co-culture with MSCs, suggesting that MSCs release factors, which promote proliferation and inhibit apoptosis. This is in line with previous studies in animal models or in vitro systems using animal cells, where MSCs were shown to rescue radiotherapy- or chemotherapy-mediated damage in organs, such as the testis, colon, or liver [56–59]. However, the direct anti-apoptotic and pro-proliferative effect of MSCs was only demonstrated in TM4 mouse Sertoli cell line in vitro so far. In in vivo studies the contribution of other cell types, such as immune cells, to the anti-apoptotic and/or pro-proliferative outcome of MSC treatment could not have been excluded [57, 58]. Our results demonstrate that long-term culture of small intestine organoids after treatment with busulfan decreases both the size and the number of organoids. The reduced number of organoids might be a consequence of an oxidative stress induction by busulfan [54], which results in a delayed increase in cell death. However, the late adverse effect of other pathways activated by busulfan cannot be excluded. Importantly, co-culture with MSCs was also beneficial in counteracting these long-term detrimental effects of busulfan. Both size and number of organoids increased in the presence of MSCs, suggesting that they have long-lasting anti-apoptotic and pro-proliferative activity.

As mentioned above, our proteomic and transcriptomic analysis indicated that MSCs revert the effect of busulfan on the expression of genes regulating proliferation and apoptosis in small intestine epithelium. Although we detected the same processes as being differentially regulated on both transcriptome and proteome level, the distinctly affected genes had only partial to little overlap. This could be due to the timing of the analysis and is not uncommon when comparing proteomic and transcriptomic data [60]. It is likely that for some proteins more time following busulfan and MSC treatment is required to detect changes in their expression.

Another interesting group of genes, which stood out as affected by busulfan and reversely regulated by co-culture with MSCs, were genes controlling the EMT pathway. EMT is an essential event during organ development, plays a key role in wound healing and tissue regeneration, and is important during cancer development and metastasis [61]. The upregulation of established EMT regulators, such as SNAI2, VIM, TGFBI, FGF2, and MMP2 by busulfan could indicate the pro-carcinogenic long-term effects of this chemotherapeutic, which correspond to its DNA alkylating activity [62]. However, the upregulation of EMT pathway could also indicate a response of the intestinal epithelium to the damage induced by busulfan and the activation of the healing program. Likewise, EMT genes induced by MSCs in intestinal epithelium damaged by busulfan, e.g. MMP2, FN1, or COL1A1 could also serve the same purpose of starting the regeneration program. However, within the genes that were specifically regulated by MSCs in busulfan-treated intestinal epithelium and not in the control organoids, there are only two genes with a demonstrated link to EMT. These genes, IKZF and PCK1, are not classical EMT regulators and from the limited evidence available they seem to have opposite functions in the control of EMT [63, 64]. Similarly, the proteomic regenerative signature of MSCs specific to the rescue of busulfan-mediated damage in intestinal epithelium does not include proteins regulating EMT and contains only the factors governing proliferation and apoptosis pathways. Therefore, the role of EMT in the regenerative effect of MSCs on busulfan-mediated damage in intestinal epithelium is not completely clear and requires further investigation. It is possible that the timing of our analysis is not optimal and verifying the expression of genes and proteins regulating the EMT pathway at different time points would give a better insight into its role in the MSC-mediated rescue.

Our functional analysis of proliferation and apoptosis indicate that MSCs have a general anti-apoptotic and pro-proliferative effect on intestinal epithelium. In the control organoids they inhibit naturally occurring apoptosis and albeit not statistically significant, MSCs also promote intestinal epithelium proliferation in control cells (Additional file 1: Supplementary Figs. S6 and S7 respectively). Nevertheless, they do exhibit a specific rescue effect on small intestine epithelium damaged by busulfan, as indicated by a distinct proteomic regenerative signature detected in our analysis. To get a better molecular insight into this MSC-mediated regeneration, it would be especially interesting to further investigate the DRG1 protein from the regenerative signature of MSCs. DRG1 expression is downregulated by MSCs in control, but upregulated in busulfan-treated intestinal organoids, which suggests that its role in the effect of MSC on intestinal epithelium may change depending on the condition of the cells and the environmental context. DRG1 is a highly conserved GTP-binding protein involved in the regulation of cell proliferation, translation, and microtubules [65]. Recently, it has been also shown to control epithelial cell–cell junctions and intestinal maturation, which illustrates its multifunctional potential [66].

There is growing evidence that MSCs execute their beneficial effects through paracrine signaling [15, 67, 68]. In our model, MSCs were cultured around the matrigel drop containing small intestinal organoids, so there was no stringent separation of the two cell types. Although we have not seen a very clear physical interaction between MSCs and organoids, we cannot exclude that cell–cell contact did play a role in the intercellular communication. Our data from co-culture experiments in the Transwell system, where organoids and MSCs were separated by a membrane, indicate that MSC secretome does contribute to the demonstrated rescue effect. More detailed imaging analysis of our co-culture model and the secretome of MSCs in these cultures is necessary to better understand the molecular nature of the MSCs regenerative signaling.

The co-culture system developed in this study with small adjustments can potentially also be used more broadly and outside of GvHD context, to study recovery from different sorts of damage, e.g. irradiation, and in different types of cancer. Likewise, it can serve to investigate an interplay between cancer cells and their environment, as MSCs are known to be important components of the tumor niche. Another advantage of this model system is that it can be relatively easily scaled up, automated, and used in a high-throughput fashion. This will allow to screen more MSC donors, patient derived organoid types, and opens the door for personal medicine approach.

## Conclusions

In conclusion, the MSC organoid co-culture model developed in this study is a valuable tool to investigate molecular details of communication between MSCs and small intestine epithelium. Our model allows the investigation of this interplay between MSC and small intestine epithelium in an environment that mimics more closely the in vivo situation of chemotherapy conditioning than currently available in vitro systems and at the same time offers a lower complexity than animal models.

### Supplementary Information

**Additional file 1.** **Supplementary Fig. S1.** Efficacy of MSC treatment in the in vitro co-culture model of small intestine organoids and MSCs. A. Co-culture with 50.000 MSCs increased the size of healthy small intestine organoids in organoid donor 1, but did not affect the size of organoid donor 2. B. Co-culture with MSCs did not affect the number of healthy and busulfan-treated organoids. C. Representative images of healthy and busulfan-treated organoids co-cultured with an effective MSC donor (donor 8). D. The quantification of the size of these organoids at 48 h after co-culture is shown. E. Representative images of co-cultures of healthy organoids and busulfan-treated organoids with a not effective MSC donor (donor 6). F. The quantification of the size of these organoids at 48 h after co-culture. The quantification of surface area of the organoids and the number of organoids was represented as fold change as compared to control. Results are shown as means ± SEM of data from 2 different organoid donors co-cultured with at least 3 MSC donors. Due to the large biologic variation in organoid size, the statistical analysis of the effect of individual MSC donors (D and F) on the size of control and busulfan treated organoids was based on all evaluable individual organoids (of at least 3 organoid/matrigel droplets cultured in different wells). Scale bars, 1000 μm. * p <0.05, ** p <0.01, *** p <0.001, and **** p <0.0001 as compared to control (Kruskal-Wallis test or a Mann Whitney test). **Supplementary Fig. S2.** Effects of MSC treatment on the size and number of healthy organoids at 7 days after coculture. A. Co-culturing MSCs with healthy organoids increased the size of organoid donor 1 and organoid donor 2 at 7 days after MSC treatment. B. Co-culturing MSCs with healthy organoids did not affect the number of organoids in organoid donor 1. The quantification of surface area of the organoids and the number of organoids was represented as fold change as compared to control. Results are shown as means ± SEM of data from 2 different organoid donors co-cultured with at least 3 MSC donors. * p <0.05 as compared to control (Kruskal-Wallis test). **Supplementary Fig. S3.** Transcriptomic analysis of healthy small intestine organoids co-cultured with MSCs. A. DE analysis of genes in healthy small intestine organoids co-cultured with 10.000 MSCs and without MSCs (red dots indicate significant genes; p value <0.05) and the accompanying upregulated (B) and downregulated (C) pathways are shown. **Supplementary Fig. S4.** Proteomic analysis of healthy small intestine organoids co-cultured with MSCs. A. DE analysis of proteins in healthy small intestine organoids co-cultured with 10.000 MSCs and without MSCs (red dots indicate significant proteins; adjusted p value ≤ 0.1 and ≥ 1.5-fold change differences) and the accompanying upregulated (B) and downregulated (C) pathways are shown. **Supplementary Fig. S5.** Proteomic analysis of busulfan-treated small intestine organoids co-cultured with and without MSCs. The accompanying upregulated (A) and downregulated (B) pathways of the DE analysis between busulfan-treated organoids co-cultured with 10.000 MSCs and without MSCs (Fig. 6A). C. Heat map of the differentially expressed proteins involved in the EMT, proliferation, and apoptosis pathway in busulfan-treated organoids co-cultured with and without MSCs is shown (N=3). **Supplementary Fig. S6.** Effects of MSC treatment on the apoptosis of healthy small intestine organoids. A. Gating strategy of single cell organoids in flow cytometry as used in Fig. 7A, Fig. 7B, and supplementary Fig. S6B. B. Effects of co-culture of healthy organoids with 0, 5.000, 10.000, and 50.000 MSCs on the percentage of apoptotic single cell organoids are shown. Results are shown as means ± SEM of organoid donor 1 and organoids donor 2 co-cultured with at least 3 MSC donors. * p <0.05 as compared to control (Kruskal-Wallis test). **Supplementary Fig. S7.** Effects of MSC treatment on the proliferation of healthy small intestine organoids. A Gating strategy of single cell organoids in flow cytometry as used in Fig. 7C, Fig. 7B, and supplementary Fig. S7C. B. Effect of co-culturing healthy organoids with 0, 5.000, 10.000, and 50.000 MSCs on the percentage of proliferating single cell organoids is shown. Results are shown as means ± SEM of organoid donor 1 and organoids donor 2 cocultured with at least 3 MSC donors. C. Effect of co-culturing healthy organoids with 0, 5.000, 10.000, and 50.000 MSCs on the percentage of viable MSCs is shown. Results are shown as means ± SEM. **Supplementary Table S1.** Effect of co-culture with different bone marrow-derived MSC donors on size of organoids damaged with busulfan # Fold change of the measured surface area of busulfan-treated organoids co-cultured with MSCs as compared to organoids co-cultured without MSCs.

## Data Availability

The raw datasets generated and/or analysed during the current study will be made available upon request to the corresponding author. RNA sequencing data have been deposited at the European Genome-phenome Archive (EGA), which is hosted by the EBI and the CRG, under accession number EGAS00001007432. Mass spectrometry proteomics data have been deposited to the ProteomeXchange Consortium via the PRIDE partner repository with the dataset identifier PXD034822.

## Abbreviations

HSCT: Allogenic hematopoietic stem cell transplantation
aGvHD: Acute graft-versus-host disease
MSCs: Mesenchymal stromal/stem cells
EMT: Epithelial to mesenchymal transition
mTORC1: Mammalian target of rapamycin 1
KRAS: Kirsten rat sarcoma viral oncogene
SNAI2: Snail family transcriptional repressor 2
VIM: Vimentin
TGFBI: Transforming growth factor beta induced
FGF2: Fibroblast growth factor 2
MMP2: Matrix metalloproteinase-2
FN1: Fibronectin 1
COL1A1: Collagen 1 a
IKZF: Ikaros zinc finger
PCK1: Phosphoenolpyruvate carboxykinase 1
DRG1: Developmentally regulated GTP binding protein 1

## References

[1] Copelan EA (2006). Hematopoietic stem-cell transplantation. N Engl J Med.

[2] Jagasia M (2012). Risk factors for acute GVHD and survival after hematopoietic cell transplantation. Blood.

[3] Ramachandran V, Kolli SS, Strowd LC (2019). Review of graft-versus-host disease. Dermatol Clin.

[4] Jansen SA, et al. Chemotherapy-induced intestinal injury promotes Galectin-9-driven modulation of T cell function. bioRxiv. 2023. 10.1016/j.isci.2024.110072 PMC11176658 38883813

[5] Blazar BR, Murphy WJ, Abedi M (2012). Advances in graft-versus-host disease biology and therapy. Nat Rev Immunol.

[6] Westin JR (2011). Steroid-refractory acute GVHD: predictors and outcomes. Adv Hematol.

[7] Axt L (2019). Retrospective single center analysis of outcome, risk factors and therapy in steroid refractory graft-versus-host disease after allogeneic hematopoietic cell transplantation. Bone Marrow Transplant.

[8] Macmillan ML (2007). A phase 2/3 multicenter randomized clinical trial of ABX-CBL versus ATG as secondary therapy for steroid-resistant acute graft-versus-host disease. Blood.

[9] Mannina D, Kröger N (2019). Janus kinase inhibition for graft-versus-host disease: current status and future prospects. Drugs.

[10] Kelly K, Rasko JEJ (2021). Mesenchymal stromal cells for the treatment of graft versus host disease. Front Immunol.

[11] Zeiser R (2020). Ruxolitinib for glucocorticoid-refractory acute graft-versus-host disease. N Engl J Med.

[12] Galderisi U, Peluso G, Di Bernardo G (2022). Clinical trials based on mesenchymal stromal cells are exponentially increasing: where are we in recent years?. Stem Cell Rev Rep.

[13] Kim N, Cho SG (2013). Clinical applications of mesenchymal stem cells. Korean J Intern Med.

[14] da Silva Meirelles L, Chagastelles PC, Nardi NB (2006). Mesenchymal stem cells reside in virtually all post-natal organs and tissues. J Cell Sci.

[15] Varderidou-Minasian S, Lorenowicz MJ (2020). Mesenchymal stromal/stem cell-derived extracellular vesicles in tissue repair: challenges and opportunities. Theranostics.

[16] Spees JL, Lee RH, Gregory CA (2016). Mechanisms of mesenchymal stem/stromal cell function. Stem Cell Res Ther.

[17] Te Boome LC (2015). Biomarker profiling of steroid-resistant acute GVHD in patients after infusion of mesenchymal stromal cells. Leukemia.

[18] Sato T (2011). Long-term expansion of epithelial organoids from human colon, adenoma, adenocarcinoma, and Barrett’s epithelium. Gastroenterology.

[19] Takashima S (2019). T cell-derived interferon-γ programs stem cell death in immune-mediated intestinal damage. Sci Immunol.

[20] Dominici M (2006). Minimal criteria for defining multipotent mesenchymal stromal cells. The International Society for Cellular Therapy position statement. Cytotherapy.

[21] Vonk LA (2018). Mesenchymal stromal/stem cell-derived extracellular vesicles promote human cartilage regeneration in vitro. Theranostics.

[22] Baak LM (2022). Feasibility and safety of intranasally administered mesenchymal stromal cells after perinatal arterial ischaemic stroke in the Netherlands (PASSIoN): a first-in-human, open-label intervention study. Lancet Neurol.

[23] Prins HJ (2009). Bone-forming capacity of mesenchymal stromal cells when cultured in the presence of human platelet lysate as substitute for fetal bovine serum. Tissue Eng Part A.

[24] van der Wagen LE (2020). Efficacy of MSC for steroid-refractory acute GVHD associates with MSC donor age and a defined molecular profile. Bone Marrow Transplant.

[25] Lindemans CA (2015). Interleukin-22 promotes intestinal-stem-cell-mediated epithelial regeneration. Nature.

[26] Hashimshony T (2016). CEL-Seq2: sensitive highly-multiplexed single-cell RNA-Seq. Genome Biol.

[27] Li H, Durbin R (2010). Fast and accurate long-read alignment with Burrows-Wheeler transform. Bioinformatics.

[28] Love MI, Huber W, Anders S (2014). Moderated estimation of fold change and dispersion for RNA-seq data with DESeq2. Genome Biol.

[29] Chen EY (2013). Enrichr: interactive and collaborative HTML5 gene list enrichment analysis tool. BMC Bioinform.

[30] Ruijter JM (2006). Factor correction as a tool to eliminate between-session variation in replicate experiments: application to molecular biology and retrovirology. Retrovirology.

[31] Iwamoto T (2004). DNA intrastrand cross-link at the 5′-GA-3′ sequence formed by busulfan and its role in the cytotoxic effect. Cancer Sci.

[32] Kaur H, Moreau R (2019). Role of mTORC1 in intestinal epithelial repair and tumorigenesis. Cell Mol Life Sci.

[33] Wang P, Li X, Xie Y (2020). B4GalT1 regulates apoptosis and autophagy of glioblastoma in vitro and in vivo. Technol Cancer Res Treat.

[34] Ito K (2011). The role of the CNOT1 subunit of the CCR4-NOT complex in mRNA deadenylation and cell viability. Protein Cell.

[35] Lu X (2012). The membrane topological analysis of 3β-hydroxysteroid-Delta24 reductase (DHCR24) on endoplasmic reticulum. J Mol Endocrinol.

[36] Lu L (2016). DRG1 is a potential oncogene in lung adenocarcinoma and promotes tumor progression via spindle checkpoint signaling regulation. Oncotarget.

[37] Miettinen TP (2018). Thermal proteome profiling of breast cancer cells reveals proteasomal activation by CDK4/6 inhibitor palbociclib. Embo J.

[38] Yin Y (2018). The function and clinical significance of eIF3 in cancer. Gene.

[39] Ren H (2022). Matrine impedes colorectal cancer proliferation and migration by downregulating endoplasmic reticulum lipid raft associated protein 1 expression. Bioengineered.

[40] Zhu J (2020). HIP1R acts as a tumor suppressor in gastric cancer by promoting cancer cell apoptosis and inhibiting migration and invasion through modulating Akt. J Clin Lab Anal.

[41] Hu Y (2021). IFI35 is involved in the regulation of the radiosensitivity of colorectal cancer cells. Cancer Cell Int.

[42] Chen Y (2020). Interleukin 1β/1RA axis in colorectal cancer regulates tumor invasion, proliferation and apoptosis via autophagy. Oncol Rep.

[43] Hahn H (2020). Structural insight on functional regulation of human MINERVA protein. Int J Mol Sci.

[44] Qi J (2020). Comprehensively analyzed macrophage-regulated genes indicate that PSMA2 promotes colorectal cancer progression. Front Oncol.

[45] Song S (2021). Reticulon 3-mediated Chk2/p53 activation suppresses hepatocellular carcinogenesis and is blocked by hepatitis B virus. Gut.

[46] Trotta AP (2013). Knockdown of the cochaperone SGTA results in the suppression of androgen and PI3K/Akt signaling and inhibition of prostate cancer cell proliferation. Int J Cancer.

[47] Lin M, Jiang M, Ding F, Cao Z (2017). Syntaxin-4 and SNAP23 act as exocytic SNAREs to release NGF from cultured Schwann cells. Neurosci Lett.

[48] Pan X (2022). Circular RNA circ-TNPO3 inhibits clear cell renal cell carcinoma metastasis by binding to IGF2BP2 and destabilizing SERPINH1 mRNA. Clin Transl Med.

[49] Usukura K (2013). Tripeptidyl peptidase II in human oral squamous cell carcinoma. J Cancer Res Clin Oncol.

[50] Leboeuf D (2020). Downregulation of the Arg/N-degron pathway sensitizes cancer cells to chemotherapy in vivo. Mol Ther.

[51] Maffioli E (2017). Proteomic analysis of the secretome of human bone marrow-derived mesenchymal stem cells primed by pro-inflammatory cytokines. J Proteomics.

[52] Ohira T (2014). Systemic histopathology of infant rats exposed to busulfan. J Toxicol Pathol.

[53] Zhao L (2023). Mechanisms underlying impaired spermatogenic function in orchitis induced by busulfan. Reprod Toxicol.

[54] Rostami A (2022). Ellagic acid effects on testis, sex hormones, oxidative stress, and apoptosis in the relative sterility rat model following busulfan administration. BMC Complement Med Ther.

[55] Bartelink IH (2016). Association of busulfan exposure with survival and toxicity after haemopoietic cell transplantation in children and young adults: a multicentre, retrospective cohort analysis. Lancet Haematol.

[56] Moussa L (2020). BMP antagonists secreted by mesenchymal stromal cells improve colonic organoid formation: application for the treatment of radiation-induced injury. Cell Transplant.

[57] Lu J (2021). Human placental mesenchymal stem cells ameliorate chemotherapy-induced damage in the testis by reducing apoptosis/oxidative stress and promoting autophagy. Stem Cell Res Ther.

[58] Qian C (2020). Human amnion mesenchymal stem cells restore spermatogenesis in mice with busulfan-induced testis toxicity by inhibiting apoptosis and oxidative stress. Stem Cell Res Ther.

[59] Nicolay NH, Lopez Perez R, Debus J, Huber PE (2015). Mesenchymal stem cells—A new hope for radiotherapy-induced tissue damage?. Cancer Lett.

[60] Ghazalpour A (2011). Comparative analysis of proteome and transcriptome variation in mouse. PLoS Genet.

[61] Yang J (2020). Guidelines and definitions for research on epithelial-mesenchymal transition. Nat Rev Mol Cell Biol.

[62] Debnath P, Huirem RS, Dutta P, Palchaudhuri S (2022). Biosci Rep.

[63] Liu R (2021). O-GlcNAc modified-TIP60/KAT5 is required for PCK1 deficiency-induced HCC metastasis. Oncogene.

[64] Tian H (2017). Downregulation of AZGP1 by Ikaros and histone deacetylase promotes tumor progression through the PTEN/Akt and CD44s pathways in hepatocellular carcinoma. Carcinogenesis.

[65] Westrip CAE (2021). Developmentally regulated GTPases: structure, function and roles in disease. Cell Mol Life Sci.

[66] Lu L (2021). DRG1 maintains intestinal epithelial cell junctions and barrier function by regulating RAC1 activity in necrotizing enterocolitis. Dig Dis Sci.

[67] Noiseux N (2006). Mesenchymal stem cells overexpressing Akt dramatically repair infarcted myocardium and improve cardiac function despite infrequent cellular fusion or differentiation. Mol Ther.

[68] Iso Y (2007). Multipotent human stromal cells improve cardiac function after myocardial infarction in mice without long-term engraftment. Biochem Biophys Res Commun.

