# Supplementary material for: Mesenchymal stromal/stem cells promote intestinal epithelium regeneration after chemotherapy-induced damage
Source: Stem Cell Res Ther. 2024 Apr 29;15:125. doi: 10.1186/s13287-024-03738-9 (PMC11057078; doi:10.1186/s13287-024-03738-9)
Supplement: Supplementary file 1 — Additional file 1. Supplementary Fig. S1. Efficacy of MSC treatment in the in vitro co-culture model of small intestine organoids and MSCs. A. Co-culture with 50.000 MSCs increased the size of healthy small intestine organoids in organoid donor 1, but did not affect the size of organoid donor 2. B. Co-culture with MSCs did not affect the number of healthy and busulfan-treated organoids. C. Representative images of healthy and busulfan-treated organoids co-cultured with an effective MSC donor (donor 8). D. The quantification of the size of these organoids at 48 h after co-culture is shown. E. Representative images of co-cultures of healthy organoids and busulfan-treated organoids with a not effective MSC donor (donor 6). F. The quantification of the size of these organoids at 48 h after co-culture. The quantification of surface area of the organoids and the number of organoids was represented as fold change as compared to control. Results are shown as means ± SEM of data from 2 different organoid donors co-cultured with at least 3 MSC donors. Due to the large biologic variation in organoid size, the statistical analysis of the effect of individual MSC donors (D and F) on the size of control and busulfan treated organoids was based on all evaluable individual organoids (of at least 3 organoid/matrigel droplets cultured in different wells). Scale bars, 1000 μm. * p <0.05, ** p <0.01, *** p <0.001, and **** p <0.0001 as compared to control (Kruskal-Wallis test or a Mann Whitney test). Supplementary Fig. S2. Effects of MSC treatment on the size and number of healthy organoids at 7 days after coculture. A. Co-culturing MSCs with healthy organoids increased the size of organoid donor 1 and organoid donor 2 at 7 days after MSC treatment. B. Co-culturing MSCs with healthy organoids did not affect the number of organoids in organoid donor 1. The quantification of surface area of the organoids and the number of organoids was represented as fold change as compared to control. R [file 13287_2024_3738_MOESM1_ESM.pdf]

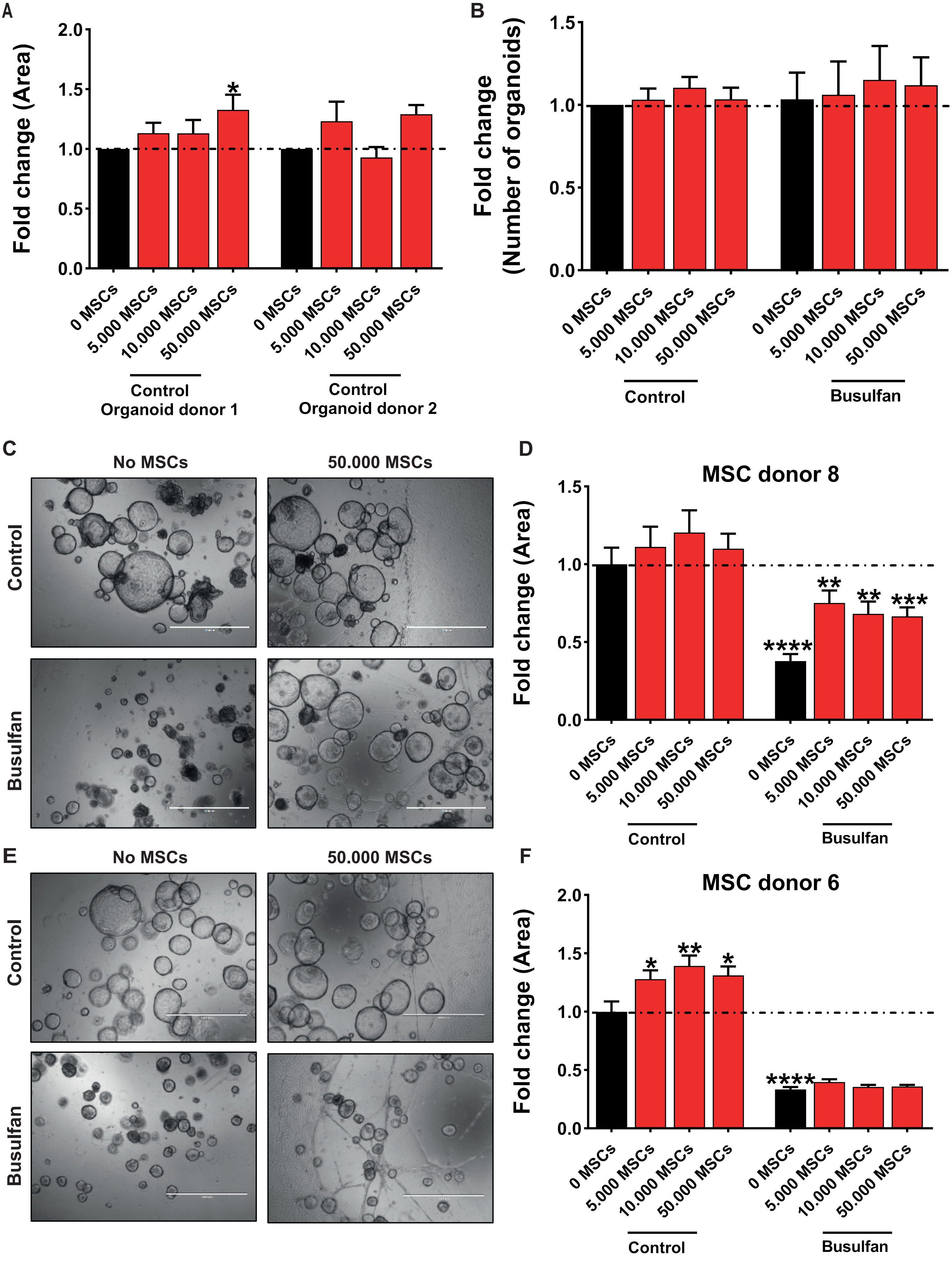

Supplementary Figure 1

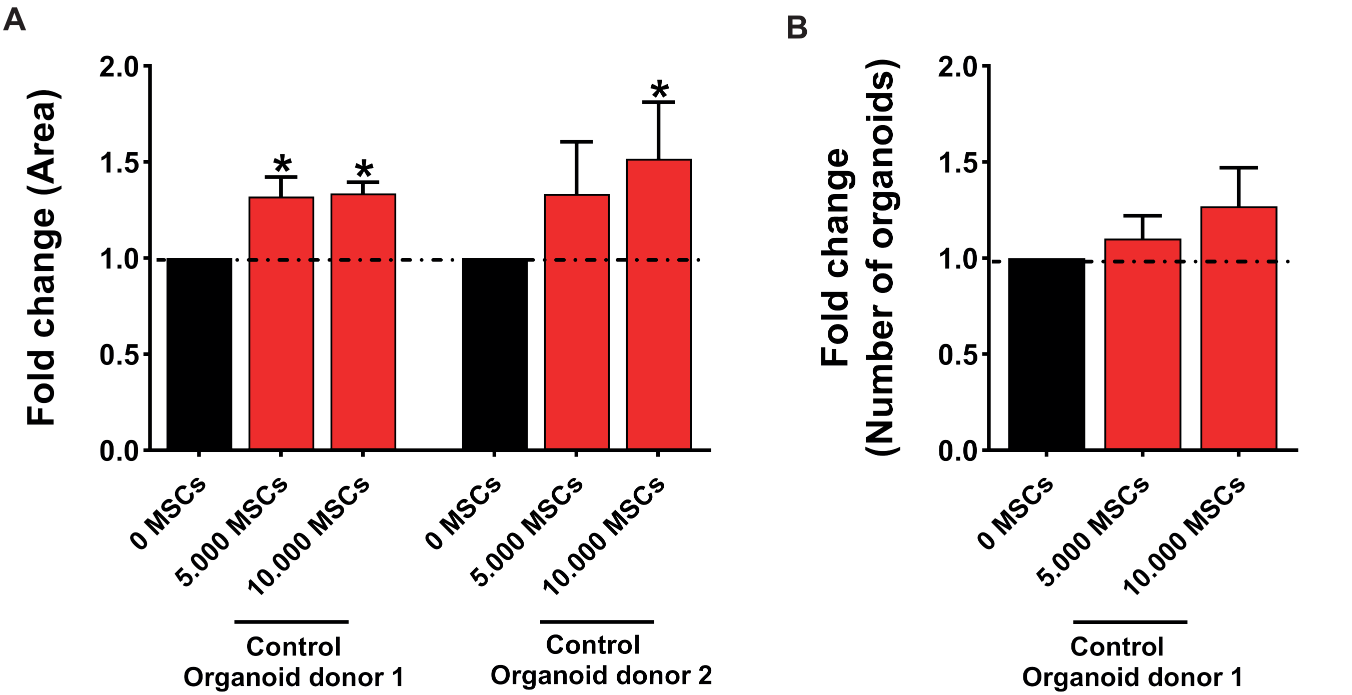

Supplementary Figure 2

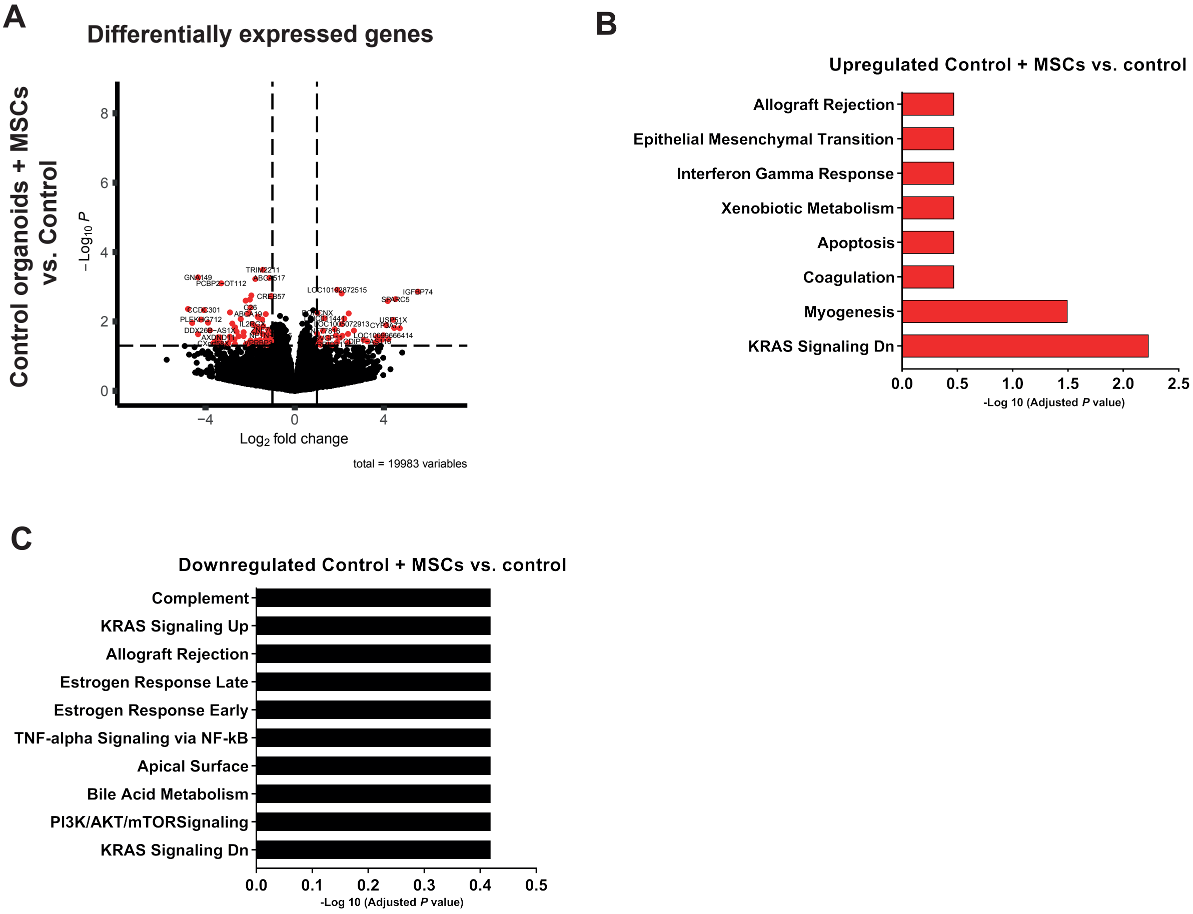

Supplementary Figure 3

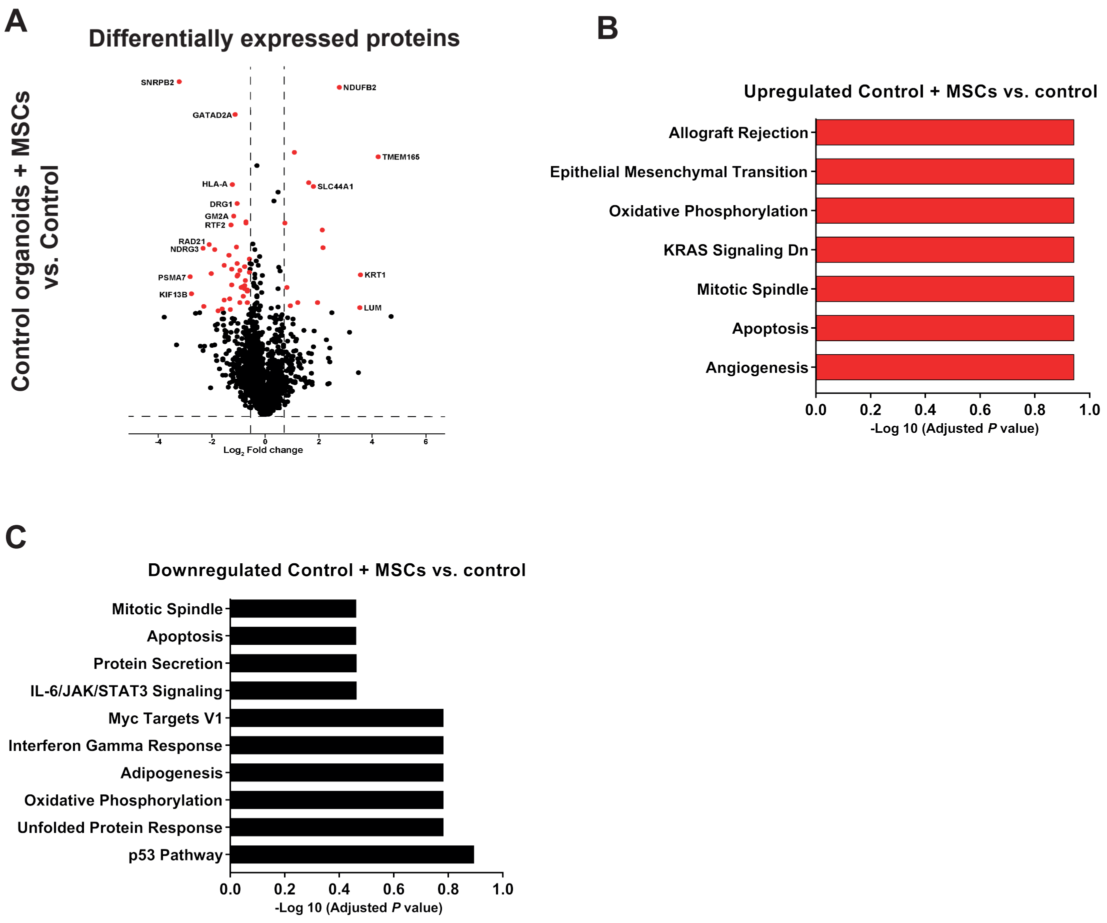

Supplementary Figure 4

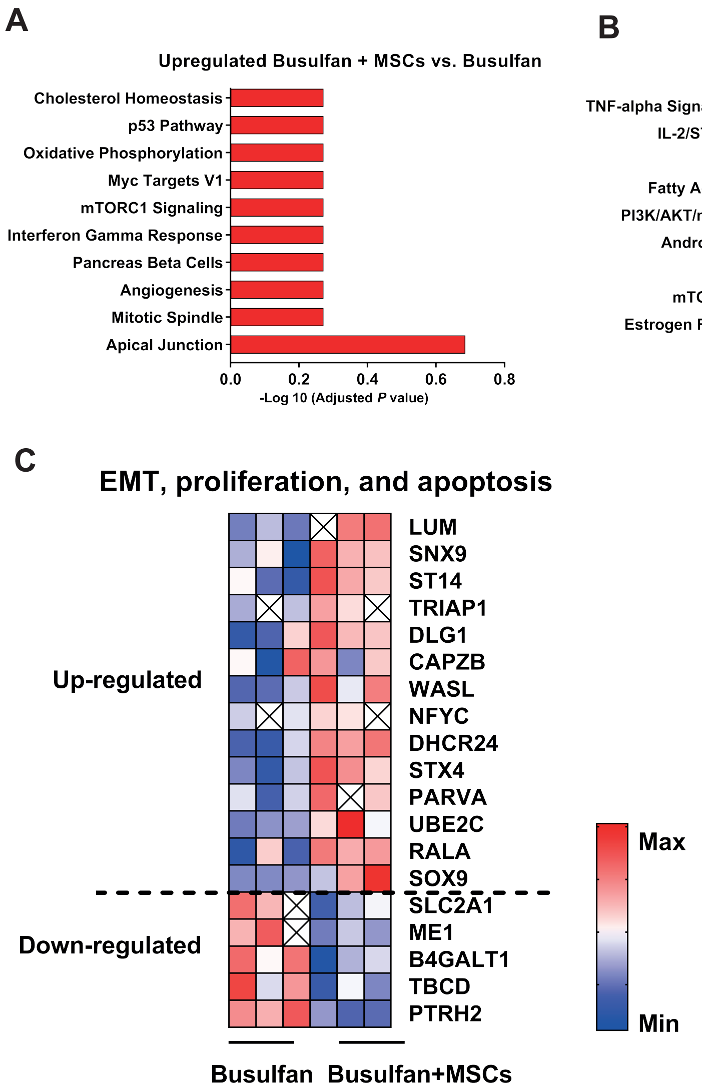

Supplementary Figure 5

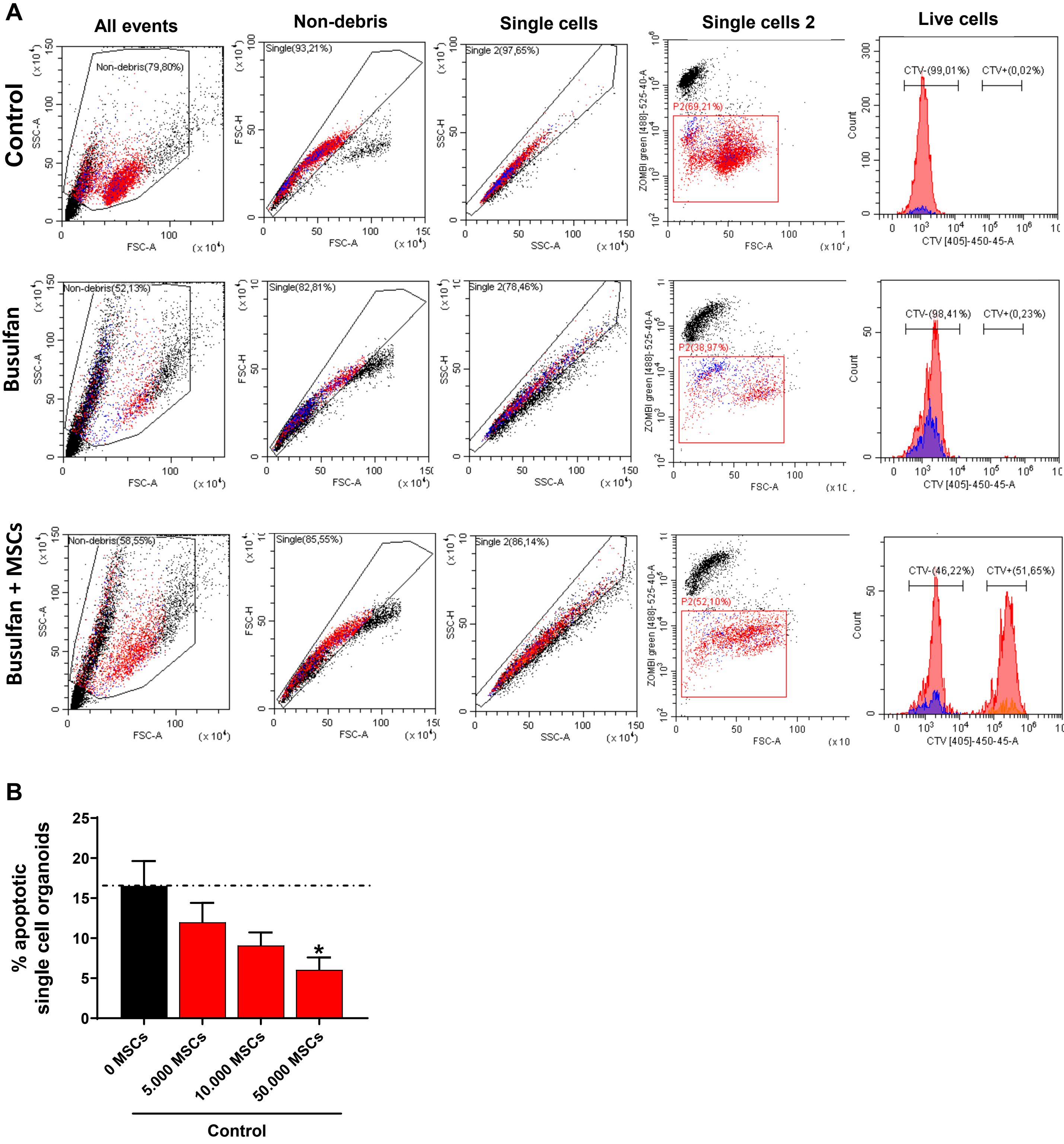

Supplementary Figure 6

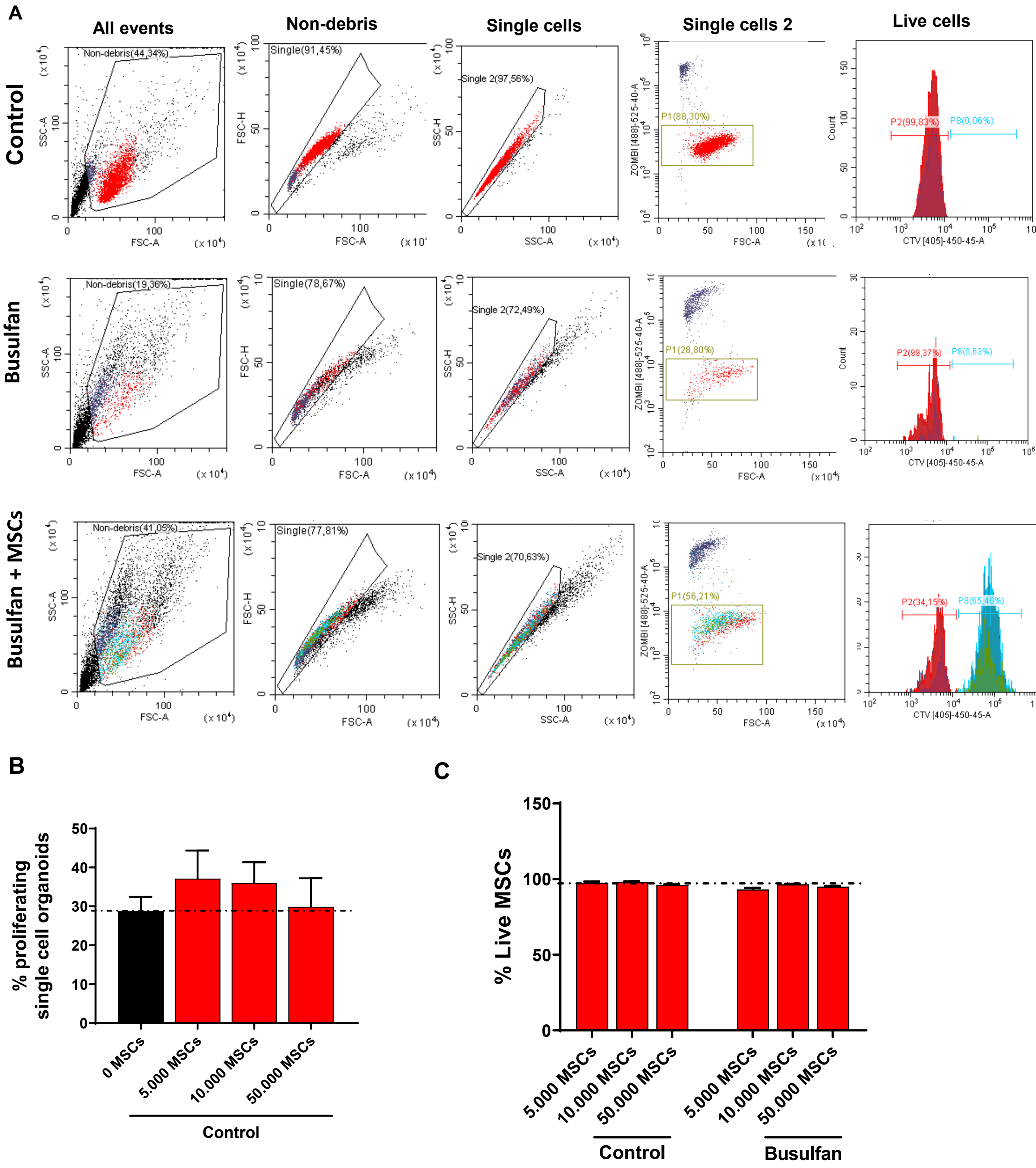

Supplementary Figure 7

Supplementary Table S1. Description and outcome of bone marrow-derived MSC donors co-cultured with busulfan-induced damaged organoids.

Organoid line	MSC donor	Age at BM donation	Gender	MSC rescue BU-induced damage (Fold change)#	p-value	
Donor 1	2	2	F	2.0	0.0010	***
Donor 1	4	14	F	1.4	0.1082	
Donor 1	5	14	F	1.6	<0.0001	****
Donor 1	6	24	M	1.2	0.1160	
Donor 1	7	26	F	1.3	0.2116	
Donor 1	8	33	M	1.6	<0.0001	****
Donor 1	16	5	X	1.2	0.0679	
Donor 1	17	14	X	1.6	<0.0001	****
Donor 1	24	2	F	1.5	<0.0001	****
Donor 2	2	2	F	1.6	0.0286	*
Donor 2	8	33	M	2.8	0.0001	***
Donor 2	16	5	X	1.7	<0.0001	****
Donor 2	24	2	F	4.6	<0.0001	****

Supplementary figure legends

Supplementary Fig. S1 Efficacy of MSC treatment in the in vitro co-culture model of small intestine organoids and MSCs. A. Co-culture with 50.000 MSCs increased the size of healthy small intestine organoids in organoid donor 1 but did not affect the size of organoid donor 2. **B.** Co-culture with MSCs did not affect the number of healthy and busulfan-treated organoids. **C.** Representative images of healthy and busulfan-treated organoids co-cultured with an effective MSC donor (donor 8). **D.** The quantification of the size of these organoids at 48 h after co-culture is shown. **E.** Representative images of co-cultures of healthy organoids and busulfan-treated organoids with a not effective MSC donor (donor 6). **F.** The quantification of the size of these organoids at 48 h after co-culture. The quantification of surface area of the organoids and the number of organoids was represented as fold change as compared to control. Results are shown as means ± SEM of data from 2 different organoid donors co-cultured with at least 3 MSC donors. Due to the large biologic variation in organoid size, the statistical analysis of the effect of individual MSC donors (D and F) on the size of control and busulfan treated organoids was based on all evaluable individual organoids (of at least 3 organoid/matrigel droplets cultured in different wells). Scale bars, 1000 µm. * $p < 0.05$, ** $p < 0.01$, *** $p < 0.001$, and **** $p < 0.0001$ as compared to control (Kruskal-Wallis test or a Mann Whitney test).

Supplementary Fig. S2 Effects of MSC treatment on the size and number of healthy organoids at 7 days after co-culture. A. Co-culturing MSCs with healthy organoids increased the size of organoid donor 1 and organoid donor 2 at 7 days after MSC treatment. **B.** Co-culturing MSCs with healthy organoids did not affect the number of organoids in organoid donor 1. The quantification of surface area of the organoids and the number of organoids was represented as fold change as compared to control. Results are shown as means ± SEM of data from 2 different organoid donors co-cultured with at least 3 MSC donors. * $p < 0.05$ as compared to control (Kruskal-Wallis test).

Supplementary Fig. S3 Transcriptomic analysis of healthy small intestine organoids co-cultured with MSCs. A. DE analysis of genes in healthy small intestine organoids co-cultured with 10.000 MSCs and without MSCs (red dots indicate significant genes; p value <0.05) and the accompanying upregulated (**B**) and downregulated (**C**) pathways are shown.

Supplementary Fig. S4 Proteomic analysis of healthy small intestine organoids co-cultured with MSCs. A. DE analysis of proteins in healthy small intestine organoids co-cultured with 10.000 MSCs and

without MSCs (red dots indicate significant proteins; adjusted p value ≤ 0.1 and ≥ 1.5-fold change differences) and the accompanying upregulated (**B**) and downregulated (**C**) pathways are shown.

Supplementary Fig. S5 Proteomic analysis of busulfan-treated small intestine organoids co-cultured with and without MSCs. The accompanying upregulated (**A**) and downregulated (**B**) pathways of the DE analysis between busulfan-treated organoids co-cultured with 10.000 MSCs and without MSCs (Fig. 6A). **C.** Heat map of the differentially expressed proteins involved in the EMT, proliferation, and apoptosis pathway in busulfan-treated organoids co-cultured with and without MSCs is shown (N=3).

Supplementary Fig. S6 Effects of MSC treatment on the apoptosis of healthy small intestine organoids. A. Gating strategy of single cell organoids in flow cytometry as used in Fig. 7A, Fig. 7B, and supplementary Fig. S6B. **B.** Effects of co-culture of healthy organoids with 0, 5.000, 10.000, and 50.000 MSCs on the percentage of apoptotic single cell organoids are shown. Results are shown as means ± SEM of organoid donor 1 and organoids donor 2 co-cultured with at least 3 MSC donors. * $p < 0.05$ as compared to control (Kruskal-Wallis test).

Supplementary Fig. S7 Effects of MSC treatment on the proliferation of healthy small intestine organoids. A. Gating strategy of single cell organoids in flow cytometry as used in Fig. 7C, Fig. 7B, and supplementary Fig. S7C. **B.** Effect of co-culturing healthy organoids with 0, 5.000, 10.000, and 50.000 MSCs on the percentage of proliferating single cell organoids is shown. Results are shown as means ± SEM of organoid donor 1 and organoids donor 2 co-cultured with at least 3 MSC donors. **C.** Effect of co-culturing healthy organoids with 0, 5.000, 10.000, and 50.000 MSCs on the percentage of viable MSCs is shown. Results are shown as means ± SEM.

Supplementary Table S1. Effect of co-culture with different bone marrow-derived MSC donors on size of organoids damaged with busulfan.

Fold change of the measured surface area of busulfan-treated organoids co-cultured with MSCs as compared to organoids co-cultured without MSCs.
